# Patient-initiated follow-up in surgical specialties: meta-analysis and meta-regression

**DOI:** 10.1093/bjsopen/zrag147

**Published:** 2026-09-21

**Authors:** Renato Pitesa, Stephanie Moody, Andrew G Hill

**Affiliations:** Department of Surgery, University of Auckland, Auckland, New Zealand; Department of General Surgery, Middlemore Hospital, Auckland, New Zealand; Department of Surgery, University of Auckland, Auckland, New Zealand; Department of General Surgery, Middlemore Hospital, Auckland, New Zealand

**Keywords:** systematic review, health services, self-referral, models of care, outpatient burden, cancer surveillance

## Abstract

**Background:**

Rising outpatient demand has accelerated adoption of patient-initiated follow-up in surgical practice. This review evaluated whether patient-initiated follow-up safely reduces clinic burden while maintaining patient outcomes across specialties.

**Methods:**

MEDLINE, Embase, and CENTRAL were searched (2014–2025) for studies of patient-initiated follow-up, defined as a model enabling clinically stable patients to self-initiate follow-up when new or concerning symptoms arise, rather than attending prearranged appointments. Primary outcomes included outpatient appointment volume and patient-reported quality of life. Where studies reported single-arm utilization data, a random-effects meta-analysis of proportions was undertaken. Exploratory meta-regression examined pathway-level factors associated with utilization. Randomized clinical trials (RCTs) were assessed using the revised Cochrane risk-of-bias 2 tool, observational cohort studies using the Newcastle–Ottawa Scale, and qualitative studies using the Critical Appraisal Skills Programme checklist.

**Results:**

Of 1776 manuscripts identified, 34 studies across 6 surgical specialties (breast, colorectal, otorhinolaryngology, orthopaedics, plastics, and urology) were included: 4 RCTs, 17 observational cohort studies, 11 qualitative studies, and 2 mixed-methods studies. Observational studies consistently reported reductions in routine outpatient attendance—including a reduction from 45·0% to 6·0% in first specialist appointments in patient-initiated follow-up in colorectal surgery (*P* < 0·001) and 52·5% of patients with high-risk cutaneous squamous cell carcinoma not recontacting the clinic—without evidence of increased adverse outcomes. RCTs demonstrated non-inferior patient-reported quality-of-life outcomes with patient-initiated *versus* standard follow-up, alongside reductions in physician contact, including a 50% reduction in planned consultations in breast cancer (1·9 *versus* 3·8 per patient; *P* < 0·001) and a reduction in specialist contacts from 42·0% to 23·0% in rectal cancer. In a meta-analysis (1806 participants), the pooled patient-initiated follow-up utilization proportion was 0·19 (95% confidence interval 0·09 to 0·36; *I*^2^ = 95%), indicating substantial context-dependent variation. In oncological settings—particularly breast, colorectal, melanoma, and head and neck cancer—structured surveillance and rapid-access systems were identified as critical safety adjuncts to implementation.

**Conclusion:**

Patient-initiated follow-up can reduce outpatient burden in selected surgical populations without compromising patient-reported outcomes; however, its effects are dependent on the clinical context and pathway design. Risk-stratified and hybrid models may be the most appropriate, particularly in oncology. Further high-quality trials and robust economic evaluations are required to define optimal implementation strategies.

## Teaser

This systematic review and meta-analysis evaluated patient-initiated follow-up (PIFU) across surgical specialties to determine its impact on outpatient utilization, safety, and patient-reported outcomes. Across 34 studies, PIFU generally reduced clinic volume without compromising patient safety or quality of life, although there was marked variation in utilization by pathway design and specialty. Structured safety-netting and hybrid models, particularly in oncological context, are likely most appropriate pending further high-quality trials with economic evaluation.

## Introduction

Traditional outpatient follow-up in surgical care relies on routine, clinician-led outpatient appointments intended to differentiate benign from sinister diagnoses, monitor postoperative recovery, and detect disease recurrence. Although essential for some patients, this model often results in unnecessary visits, contributing to increased healthcare burden, inefficient resource allocation, and prolonged wait times for first specialist appointments (FSAs)^1,2^. Many patients attend clinics despite being asymptomatic, whereas others experience delays in accessing care when symptoms arise between scheduled visits. This inefficiency exacerbates already strained outpatient services and inconveniences patients, highlighting the need for a more flexible, patient-centred approach^3^.

Patient-initiated follow-up (PIFU) offers an alternative model that shifts fixed appointment scheduling to a more flexible system, allowing patients to request follow-up only when required^3^. Already well integrated into chronic disease management where follow-up monitors easily detectable changes in symptoms, particularly in rheumatology and inflammatory bowel disease, PIFU has demonstrated reduced appointment volumes, maintained clinical safety, and improved patient satisfaction^4^. Additionally, its integration with digital health solutions, such as telemedicine, electronic patient-reported outcome (PRO) measures, and automated symptom monitoring, has the potential to enhance the efficiency and safety of surgical follow-up^5^.

Previous systematic reviews^4,5^ of PIFU have focused on oncology and chronic medical conditions, with limited emphasis on surgical care pathways. Surgical follow-up includes detection of complications, functional recovery, and cancer surveillance, which may not be suitable for a PIFU approach^1,2^. This review evaluated the effectiveness, safety, and implementation considerations of PIFU across surgical settings.

## Methods

This systematic review and meta-analysis was reported in accordance with PRISMA guidelines^6^. It was registered prospectively in the International Prospective Register of Systematic Reviews (PROSPERO: CRD42025648072)^7^.

### Search strategy

The search strategy included the databases of MEDLINE and Embase via Ovid, and CENTRAL from 1 January 2014 to 31 December 2024, with an updated search carried out on 31 December 2025. A grey literature search of Google Scholar and the Cochrane Library for Cochrane Reviews was also undertaken to find existing reviews on the topic. Filters used to conduct the search included [English language] and [Adults 18+]. The specific search strings for the databases are listed in *supplementary methods*. The search string was modified to allow its application across multiple databases, and was designed and constructed with the guidance of an information specialist.

The bibliographies of all included studies were screened manually as part of a backward and forward citation search.

### Eligibility

For the purposes of this review, PIFU was defined as any model in which clinically stable patients were discharged from FSAs or routine follow-up, and encouraged to contact the clinical service when new or concerning symptoms arose rather than attending prearranged appointments. Given that PIFU is a complex service intervention rather than a discrete clinical treatment, the inclusion of multiple study designs was necessary to capture its implementation, safety, and acceptability across multiple surgical contexts.

#### Inclusion criteria

Participants were clinically stable, low-risk adult surgical patients aged (≥ 18 years) being seen in surgical outpatient/secondary care clinics for postoperative follow-up, cancer surveillance, or FSA for both benign and malignant conditions. Interventions were PIFU models. Comparators comprised standard follow-up (SFU) models, including clinician-led follow-up, active surveillance, or routine outpatient visits. Study designs included randomized clinical trials (RCTs), observational studies (prospective and retrospective cohort studies, case–control studies), and qualitative studies evaluating patient and provider perspectives on PIFU in surgical specialties.

#### Exclusion criteria

Exclusion criteria were: studies focusing on nurse-led follow-up or exclusively clinician-led telehealth follow-up, as these retain a provider-initiated scheduling structure and do not confer the core PIFU characteristic of patient-initiated contact; editorials, commentaries, and implementation protocols; conference abstracts without sufficient information; and studies including participants not eligible or suitable for PIFU, such as those with cognitive impairment, high recurrence risk, or complex postoperative requirements.

### Study selection

Two authors independently screened search results for eligibility. A preliminary screen of the title and abstracts was then performed using Rayyan (Rayyan Systems Inc., Cambridge, Massachusetts, USA), a web tool designed to assist with study screening and selection. Duplicates and articles that were out of scope were removed. Full texts of all eligible articles were retrieved, and then reviewed independently; the search was completed with the backward and forward citation search. Discrepancies were resolved by discussion and referring to a senior author.

### Data extraction

Data extraction was completed independently by two authors. Outcomes of interest were divided into primary and secondary outcome measures. The results of each reviewer’s data extraction were verified by the second reviewer. Discrepancies were resolved by discussion and referring to a senior author.

Given the heterogeneity of PIFU models and study designs, a broad outcomes framework was adopted, and data extracted from the studies adhered to a set of predefined variables. These included study characteristics, such as study design, setting, number of participants, and patient demographics.

### Outcomes of interest

The primary outcome was the impact of PIFU on clinic volume, clinical outcomes, and patient-reported health-related quality of life (HRQoL). For the purposes of this review, clinic volume outcomes included any quantitative measure of outpatient appointment frequency, attendance rates, or service utilization. Quality-of-life outcomes included data based on any validated or non-validated instrument assessing HRQoL, satisfaction, or symptom burden. Secondary outcomes included qualitative evidence of patient and clinician perspectives, clinical safety data, and economic analyses, when available for extraction.

### Quality assessment and risk of bias

RCTs were assessed using the revised Cochrane Risk of Bias 2 (RoB2) tool for each outcome of interest^8^. Observational cohort studies were assessed using the Newcastle–Ottawa Scale^9^. Qualitative studies were assessed using the Critical Appraisal Skills Programme (CASP) checklist^10^. Mixed-methods RCTs were evaluated independently using the RoB2 tool for quantitative outcomes and the CASP checklist for qualitative outcomes. Assessment of quality and risk of bias were performed independently. Included conference articles were not assessed for quality given the limited methodological informational available. Discrepancies were resolved by discussion.

### Statistical analysis

Meta-analysis was performed using RStudio version 4.3.2 (Posit PBC, Boston, Massachusetts, USA) using the ‘meta’ package. A comparative meta-analysis was not feasible because of limited or heterogeneous data; therefore, a narrative synthesis was undertaken. Quantitative and qualitative data were analysed in parallel and grouped thematically within each surgical specialty. A separate single-arm meta-analysis of the proportion of PIFU utilization was conducted where appropriate methodologically. Proportions were logit-transformed to stabilize variances, and pooled estimates were calculated using the inverse-variance method under a random-effects model with restricted maximum likelihood (REML) estimation of between-study variance (τ^2^). The Hartung–Knapp adjustment was applied to improve the robustness of confidence intervals given the small number of studies. Individual-study confidence intervals were calculated using the Clopper–Pearson method. Statistical heterogeneity was quantified using *I*^2^, τ^2^, and Cochran’s Q statistic. In addition to pooled estimates, 95% prediction intervals were calculated to estimate the range within which the true utilization proportion of a future comparable study might lie.

Given the high degree of heterogeneity observed, exploratory random-effects meta-regression was undertaken using the metafor package. Logit-transformed proportions were regressed against pathway type (for example trauma discharge, elective discharge, injection-monitoring pathways) using REML estimation. Residual heterogeneity was assessed, and the proportion of between-study variance explained (*R*^2^) was reported. Owing to the small number of studies, meta-regression findings were interpreted as exploratory and hypothesis-generating.

## Results

### Search results

**Figure 1:**
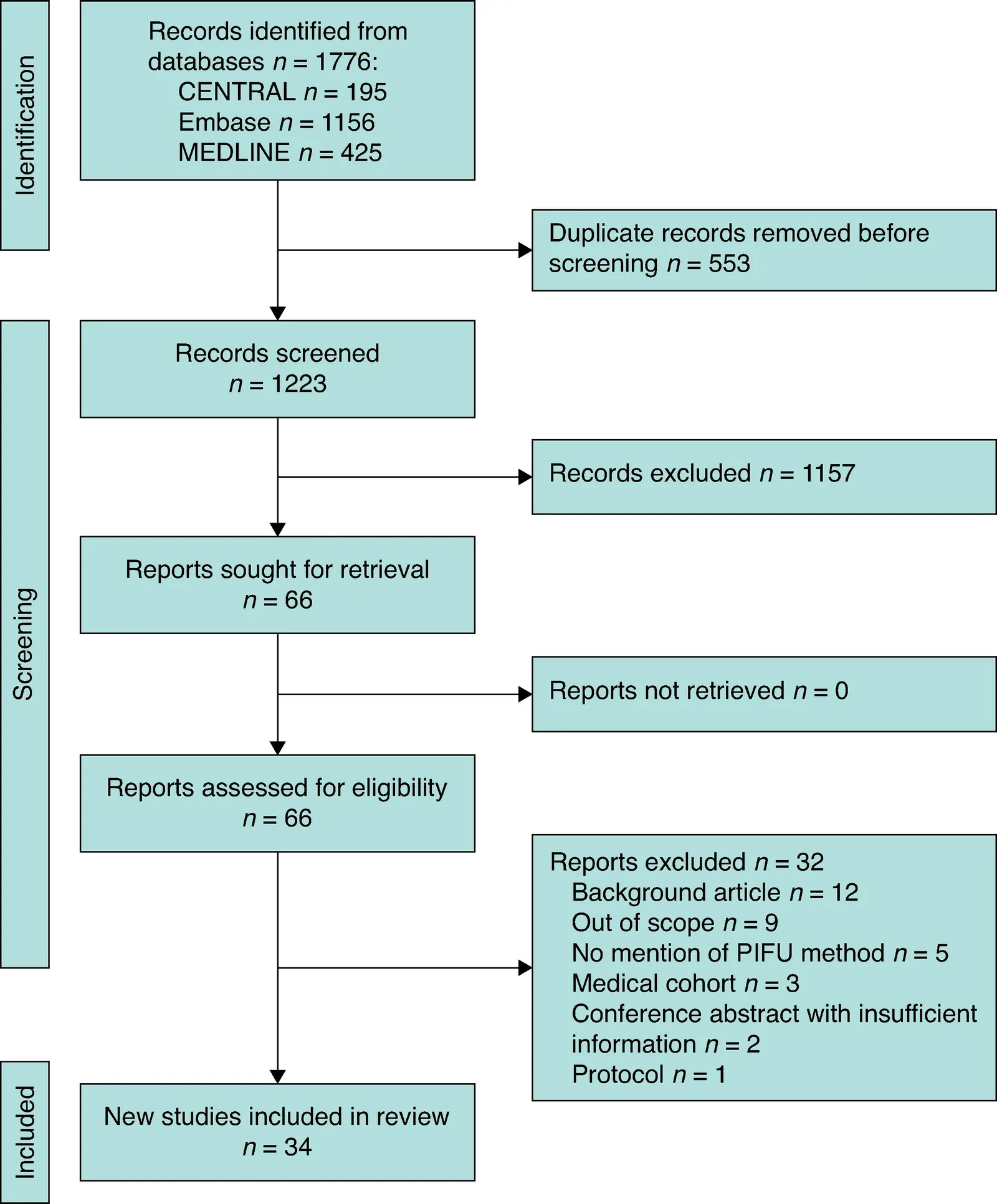
PRISMA flow diagram showing selection of articles for review PIFU, patient-initiated follow-up.

A PRISMA flow chart is shown in *Fig. 1*^6^. Thirty-four studies^11–44^ were included in this review and meta-analysis. A grey literature search of Google Scholar and the Cochrane Library identified two existing systematic reviews^4,5^ relevant to the topic; however, no additional primary studies were identified from these sources. A citation search did not reveal any additional eligible studies.

### Study characteristics

Characteristics of the studies are summarized in *Table 1*, with specific characteristics detailed in *Table S1*. Among the 34 included articles, 4 large host trials contributed multiple publications: PRAGMATIC (breast; 2 nested studies)^34,35^, FURCA (colorectal; 3 nested studies)^17,21,33^, PETNECK2 (otorhinolaryngology; 2 nested studies)^27,28^, and MEL-SELF (plastics; 5 nested studies)^22,24,25,31,39^. Where multiple publications arose from the same host trial, they were treated as independent substudies reporting distinct outcomes.

**Table 1:** Summary of study characteristics

	*n*
No. of studies	34
Publication years	2014–2025
**Study design**	
Qualitative	11
Retrospective	5
Prospective	11
Retrospective and prospective	1
Randomized clinical trial	4
Mixed-methods randomized clinical trial	2
**Country**	
Aotearoa New Zealand	1
Australia	5
Denmark	5
Netherlands	1
UK	22
**Specialty**	
Breast	9
Colorectal	6
Otorhinolaryngology	2
Orthopaedics	6
Plastics	7
Urology	4
**PIFU indication**	
Benign	8
Malignant	26

PIFU, patient-initiated follow-up.

Across the 34 included studies, patients initiated follow-up through one of two broad models: telephone contact with a clinical nurse specialist (CNS), coordinator, or direct clinic line (most common; used across all specialties); and digital platforms, including smartphone applications, web portals, or electronic symptom-reporting tools (used in MEL-SELF, PETNECK2, and several urology and colorectal studies). Several models combine both approaches.

### Quality assessment and risk of bias

**Figure 2:**
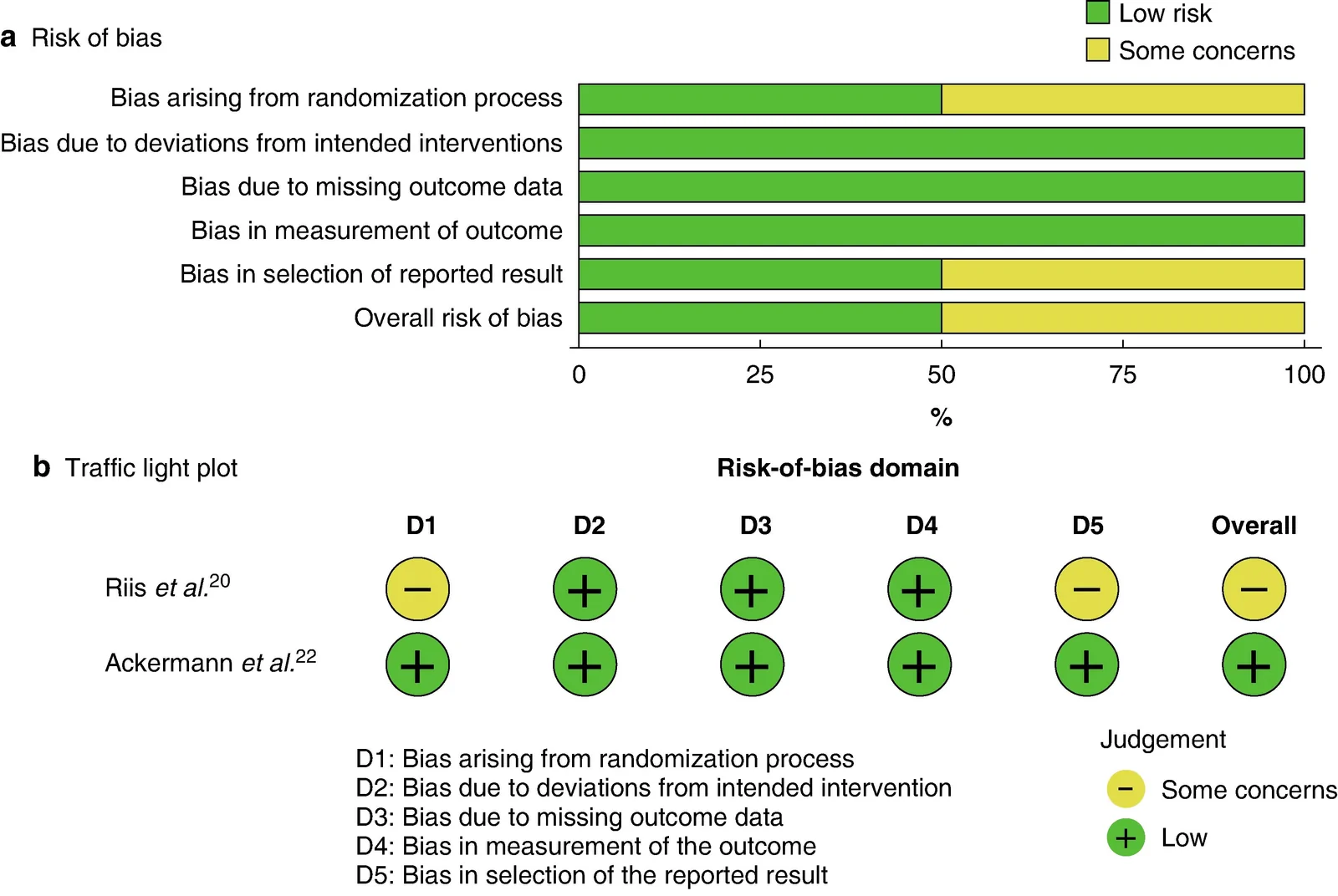
Quality evaluation of studies reporting on clinic volume **a** Cochrane risk-of-bias 2 (RoB2) summary and **b** RoB2 traffic light plot.

**Figure 3:**
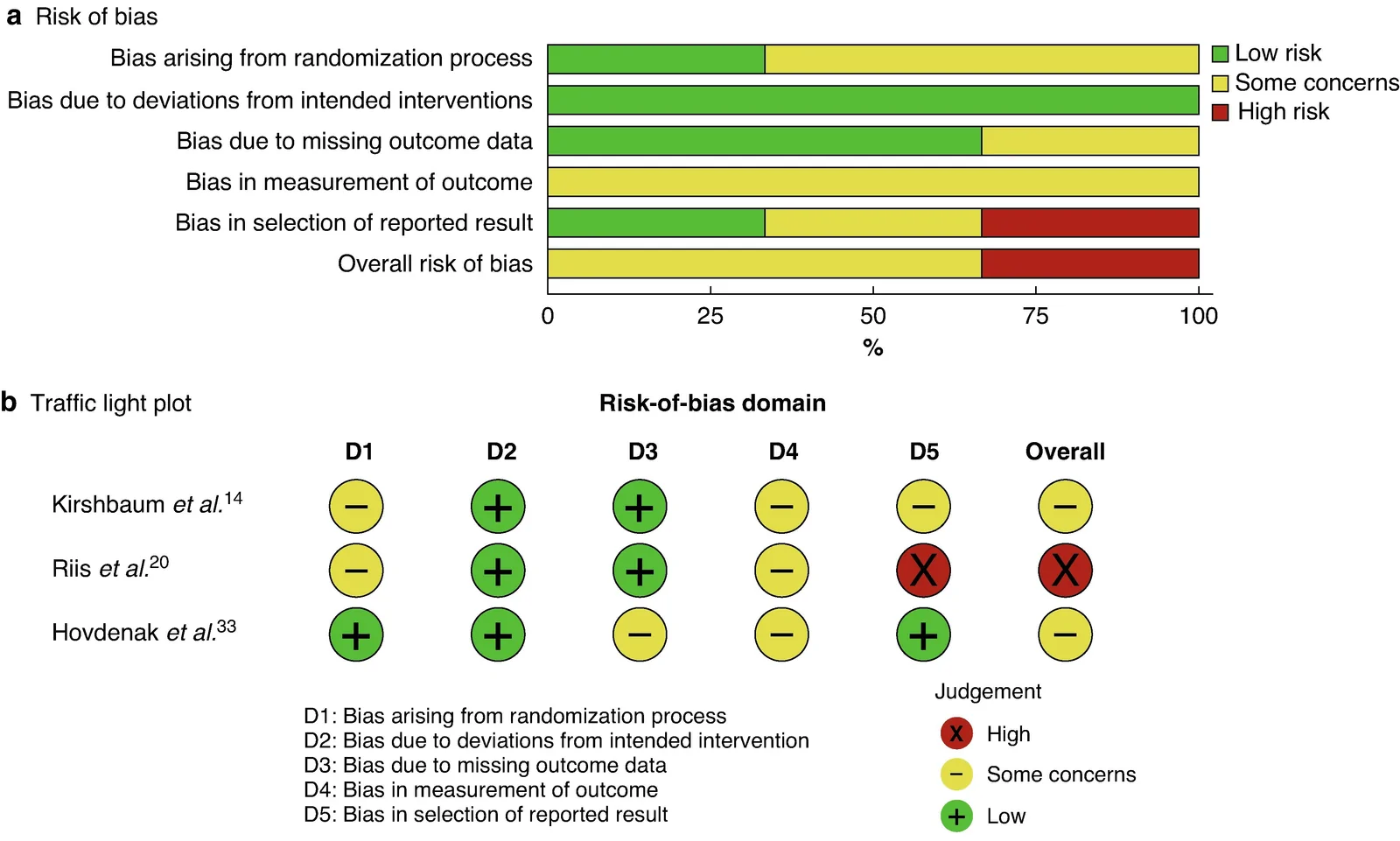
Quality evaluation of studies reporting health-related quality of life **a** Cochrane risk-of-bias 2 (RoB2) summary and **b** traffic light plot.

Four RCTs were assessed using RoB2: two full-text RCTs^14,33^ and the quantitative outcomes of two mixed-methods RCTs^20,22^. Two preliminary FURCA publications^17,21^ were not assessed independently as they report on the same trial population as the primary FURCA RCT^33^. Two studies^20,22^ reported on clinic volume outcomes (*Fig. 2*); one study^22^ was low risk in every domain, whereas the other^20^ carried some concerns in the randomization and selective-reporting domains given the lack of methodological clarity, giving an overall rating of some concerns. Three studies^14,20,33^ reported on patient-reported HRQoL and patient-reported outcomes (PROs) (*Fig. 3*); most RCTs were deemed to have some concerns, given that the outcome of interest was subjective and based on self-reports, so it was not possible to blind the investigators or participants^14,33^. One RCT^20^ was deemed to be of high risk because of unexplained missing data in selective outcome reporting and lack of sensitivity analyses to account for the effect of missingness. Across all RCTs, bias owing to deviations from intended intervention and missing data was consistently low.

Twelve full-text observational studies^13,15,23,30,34,36–38,40,42–44^ were assessed, the remaining five studies were conference abstracts excluded from formal quality assessment. The median Newcastle–Ottawa score was 6 of 9 (range 6–9), indicating moderate overall quality (*Table S2*). The comparability domain was the principal weakness, given that most studies focused on a single cohort with no comparison or control group to allow adequate adjustment for confounding. Conversely, outcome ascertainment and follow-up were strong, with only one study^13^ showing substantial attrition exceeding 20%.

Eight full-text qualitative and two mixed-methods studies^20,24,25,27–29,31,35,39,41^ were assessed. CASP appraisals were consistent across all studies, with no study failing on more than two domains (*Table S3*). All articles articulated clear aims, used appropriate qualitative designs, and provided transparent recruitment and data analysis methods. The most common shortcoming was limited reflexivity regarding how the interviewer’s positionality might have shaped data collection and interpretation.

### PIFU utilization

**Figure 4:**
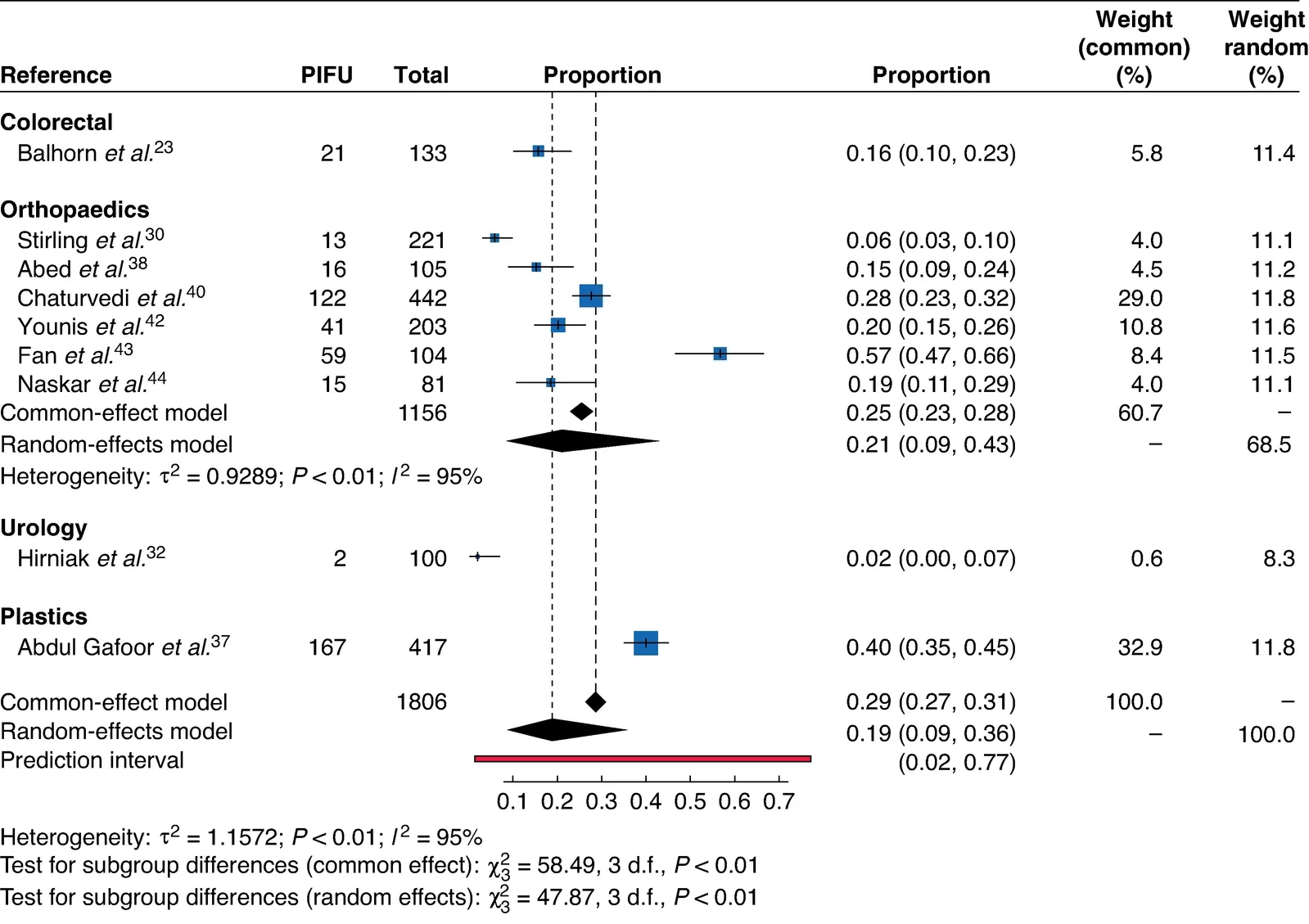
Forest plot of single-arm meta-analysis of PIFU utilization stratified by specialty The proportion of patients initiating follow-up in each cohort is shown with 95% confidence intervals. PIFU, patient-initiated follow-up.

A single-arm meta-analysis of PIFU utilization was performed across nine eligible studies from four surgical specialties (colorectal, orthopaedic, plastics, and urology). The pooled utilization proportion under a random-effects model was 0·19 (95% confidence interval (c.i.) 0·09 to 0·36), with extreme heterogeneity (*I*^2^ = 95%, τ^2^ = 1·16, *P* < 0·001) and a wide prediction interval (0·02 to 0·77), indicating substantial context-dependent variation in expected utilization across clinical contexts (*Fig. 4*). Exploratory meta-regression identified pathway type as a significant moderator; injection-monitoring pathways were associated with significantly higher utilization than discharge-based models (β = 1·94, *P* = 0·046), explaining 31·5% of between-study variance. Predicted utilization was 15·9% (95% c.i. 8·9% to 26·5%) for discharge-based pathways compared with 56·7% (18·0% to 88·7%) for injection-monitoring pathways (*I*^2^ = 95·9%).

#### Summary by specialty: breast surgery

##### PIFU method

Nine studies^11,12,14,19,20,29,34–36^ explored PIFU in breast surgery, specifically breast cancer, with two related to the PRAGMATIC study. The method of implementing PIFU was broadly consistent across all studies. Patients with early-stage breast cancer who had completed hospital treatment were advised to contact the clinic if they had any concerns or suspicions of disease recurrence and did not receive regular clinician reviews. Variations existed regarding safety netting; some centres would continue with regular mammograms either annually or every 18–24 months, or digital symptom tracking to maintain some degree of physician oversight^12,20^.

##### Patient volume

A manuscript^20^ described a significant reduction in clinic visits in patients having PIFU compared with SFU of up to 50% per patient over 2 years (mean number of consultations per patient 1·9 (95% c.i. 1·4 to 2·4) for PIFU *versus* 3·8 (3·5 to 4·1); *P* < 0·001). This reduction in patient volume did not compromise patient safety, as the PIFU group also had a smaller mean number of urgent care visits per patient compared with the SFU group (0·22 (0·09 to 0·34) *versus* 0·47 (0·29 to 0·65); *P* = 0·030). Chadha *et al*.^11^ offered PIFU to patients with benign follow-up mammograms. They evaluated 100 clinic appointments and found that 69·0% of patients had benign scans and were eligible for PIFU. Of these, 98% of the concerns voiced by patients were managed by nurse specialists without the need for an inpatient appointment. They estimated that applying these findings to their entire clinic would save 1435 clinic appointments and €251 109 annually. One study^12^ that implemented annual mammograms for 5 years found that recurrence detection was superior through PIFU compared with SFU, with 55% of recurrences identified through patient-initiated visits, 23% detected by mammography, and only 9% identified during routine clinical reviews.

##### Patient perspectives

PROs were largely positive, with one study^19^ showing that 98% of patients understood the PIFU model after an educational session and another^35^ showing that 84% were satisfied with the PIFU experience^19,35^. One report^29^ stated that most patients viewed PIFU positively, and appreciated the autonomy and flexibility of the model; however, some had concerns about accessing care, self-examination adequacy, and missing recurrence. HRQoL findings based on the European Organisation for Research and Treatment of Cancer Quality of Life Questionnaire (QLQ) C30 and QLQ-BR23 from Kirshbaum *et al*.^14^ found no significant difference in scores between the PIFU and SFU groups at any time point across 24 months of follow-up. Another study^36^ investigated whether certain socioeconomic factors of patients who had PIFU affected HRQoL, as measured by QLQ-C30 and QLQ-BR23; it was found that those in lower socioeconomic groups were not disadvantaged by PIFU, but those with a lower level of education tended to report more general breast symptoms and under-report symptoms of clinical relevance, suggesting that patient selection and health literacy is crucial for the success of PIFU.

#### Summary by specialty: colorectal surgery

##### PIFU method

Six studies^13,17,21,23,33,41^ evaluated PIFU in colorectal surgery, with cases ranging from post-colorectal cancer treatment to rectal bleeding. The FURCA trial^17,21,33^ is an ongoing RCT comparing PIFU with SFU in survivors of rectal cancer, incorporating routine surveillance computed tomography (CT) and blood testing, in addition to access to specialist consultations as needed. Other cancer PIFU models also maintained routine surveillance CT, carcinoembryonic antigen blood tests, or nurse-coordinated monitoring, ensuring a degree of clinical oversight^13,41^. Digital support systems, in addition to self-management workshops and structured education sessions, helped improve patient confidence in symptom monitoring^13^. One research paper^23^ described a PIFU model in which patients with likely benign rectal bleeding were discharged from the clinic with a contact card and advised to call the clinic if their symptoms did not resolve with conservative management. Calls were answered by a CNS and a clinic appointment was made promptly.

##### Patient volume

PIFU demonstrated a significant reduction in outpatient follow-up burden, with one RCT^21^ showing that specialist contacts were reduced from 42% in the SFU group to 23% in the PIFU group. In the same study, 57% of patients in the PIFU arm did not contact the CNS at all during the first year. Among the 43% of patients who made contact, the primary reasons were bowel dysfunction, stoma issues, pain, and psychological distress. Conversely, others found no significant differences in outpatient hospital utilization between the PIFU and SFU groups; however, those who had PIFU tended to contact the general practitioner less than those who had SFU^13^. Despite a reduction in clinic visits, clinical outcomes remained comparable, with no significant differences in recurrence rates, functional outcomes, or time to recurrence between the PIFU and SFU groups. Most recurrences were detected through routine surveillance imaging rather than patient-initiated contact^33^. Balhorn *et al*.^23^ reported that 21 patients (16·0%) used the PIFU card, making a total of 30 calls. There was a significant reduction in FSA clinic appointments between the SFU and PIFU groups (45% *versus* 6%; *P* < 0·001), and no significant difference in elective surgery rates (14% *versus* 16%, respectively). In addition, one colorectal cancer diagnosis was made in each group, suggesting no increased risk of missed cancers with PIFU.

##### Patient perspectives

PRO scores in colorectal PIFU models showed no significant differences in HRQoL at 8-month and 3-year follow-up (as measured by Functional Assessment of Cancer Therapy—Colorectal scores), or symptom burden compared with SFU, although patients in PIFU models reported greater involvement, satisfaction, and perceived support from healthcare providers, with some appreciating the reduced hospital visits, travel, and time off work^21,33,41^. Improvements in emotional well-being were observed in some studies, but these were not statistically significant^33^. One of the studies^13^ noted that patient enrolment into PIFU was possibly limited by clinician attitude and culture, and that trust over time is required for maximum clinician involvement. In addition to this, participants from one qualitative study^41^ acknowledged that PIFU would be more suited to the younger or better educated patient, especially if digital symptom and blood test monitoring was involved as it required some degree of independence and digital literacy to know when to seek clinical advice.

##### Economic value

In one article^13^, it was noted that PIFU was €165·94 more expensive per patient than SFU in the first year, which was attributable to the cost of self-management workshops and remote surveillance. Although Balhorn *et al*.^23^ did not conduct a formal cost analysis, they hypothesized that the significant reduction in clinic volume would cut costs.

#### Summary by specialty: otorhinolaryngology

##### PIFU method

PIFU in otorhinolaryngology has been evaluated predominantly for head and neck cancers. The ongoing PETNECK2 RCT is continuing to investigate this, with two qualitative articles detailing perspectives on the PIFU model from both patients and healthcare providers. The model involves patients with low-risk head and neck cancer, with normal positron emission tomography 1 year after treatment, being transitioned to PIFU. Patients will be educated about the symptoms of recurrence to monitor for, in addition to being given access to a mobile app/website and paper booklets, and, despite not being seen routinely, are guaranteed a clinic appointment within 2 weeks of contact should concerns of recurrence arise. This is in contrast to the standard care, whereby patients are reviewed every 2–6 months for 5 years after treatment to monitor for recurrence^27,28^.

##### Patient and provider perspectives

Patient and family perspectives on PIFU highlighted mixed preferences, with some appreciating the autonomy and flexibility, but others expressing concerns about losing the reassurance of routine clinical oversight. Notable concerns included challenges in self-identifying recurrence, perceived lack of expertise, and potentially unmet need for mental health support, particularly for those with pre-existing anxiety. Reliable and rapid clinician access was also considered critical to the acceptability of PIFU^28^. Among clinicians interviewed, there were ten oncologists, ten surgeons (5 otorhinolaryngological, 5 maxillofacial), eight CNSs, four speech and language therapists, one dietitian, and one research radiographer. They acknowledged the potential for improved resource allocation, but raised concerns about patient safety and the need for robust guidance on symptom recognition, especially in those susceptible to head and neck cancers, such as heavy smokers and drinkers, who may be less engaged with healthcare. Both groups emphasized that PIFU may not be for everyone, but should be supported by clear information, effective communication channels, and optional psychological support^27^.

#### Summary by specialty: orthopaedic surgery

##### PIFU method

Six studies^30,38,40,42–44^ investigated PIFU in patients with fractures and postelective hand surgery. Two studies^30,40^ described a PIFU virtual pathway for patients with a suspected scaphoid fracture that was not obvious on radiographs, discharging them from the emergency department with an information sheet, a recovery checklist, and the option to self-refer for a follow-up appointment if needed. Similarly, another study^42^ described patients with stable fracture requiring minimal follow-up who were discharged with PIFU, enabling self-initiated contact should symptoms arise rather an attending routine scheduled appointments. The remaining studies^38,43,44^ described models in which patients undergoing elective hand surgery or those with self-limiting conditions were counselled on rehabilitation, and were given a card with written and visual exercises, concerning symptoms, and a number to call if they had concerns and wanted a review.

##### PIFU utilization and further contact

Across discharged-based fracture and elective upper limb cohorts, between 72% and 86% of patients did not initiate follow-up, with no increased risk of missed fractures or complications^30,40,42^. Similarly, 82–85% of patients seen in upper limb clinic and after hand surgery did not require routine follow-up; of patients who contacted the clinic, the majority were managed successfully with corticosteroid injections, hand therapy, analgesia, and reassurance^38,44^. Some patients from the upper limb clinic cohort were referred for surgery, but this represented 6% of the total cohort (5 patients)^44^. In contrast, others^43^ reported that 56·7% of patients receiving steroid injections requested follow-up, reflecting the ongoing and fluctuating nature of symptoms in this cohort.

##### Patient perspectives

Concerns regarding patient anxiety and self-monitoring confidence were noted, particularly during the early postoperative period. Structured discharge instructions, symptom guidance, and access to specialist support mitigated these concerns^38^. Younis *et al*.^42^ reported that 80·3% (163 patients) utilizing PIFU rated the service 5 of 5, 9·9% (20) rated it 4 of 5, and the remaining 9·9% rated it 3 of 4 . Ease of use, patient autonomy, and timeliness were the most valued aspects of the service.

##### Economic value

One study^43^ reported that only 8·7% of patients requested a review within the routine 6–8-week follow-up window, whereas 91·3% did not require routine outpatient reassessment at that time point. They estimated that omitting standardized 6–8-week appointments could translate into cost savings of approximately €11 082–22 165, attributable solely to follow-up consultations being avoided.

#### Summary by specialty: plastic surgery

##### PIFU methods

Seven studies^22,24–26,31,37,39^ evaluated PIFU in plastic surgery. These were primarily in the context of melanoma and high-risk cutaneous squamous cell carcinoma (cSCC), employing self-monitoring, digital technologies, and rapid-access pathways. The MEL-SELF RCT^22^ employed total-body skin self-examinations, smartphone-compatible dermatoscope attachments for teledermoscopy, and teledermatologist review within 72 h, accompanied by expedited clinic access upon patient concern and bimonthly reminders. In high-risk cSCC, PIFU incorporated structured patient education on red-flag symptoms, direct CNS support, and guaranteed clinic access within 7 days^37^. Separately, a study^26^ on follow-up after trauma plastic surgery investigated patient-requested consultant reviews guaranteed within 3 months, as opposed to routine clinics, to reduce unnecessary clinic visits and improve attendance rates.

##### Patient volume

Variable effects on clinic volume were observed, with outcomes contingent on the model employed. In the MEL-SELF RCT^22^, the intervention cohort experienced a 1·5-fold increase in clinic visits compared with the SFU group, along with increased lesion excisions. However, this was consistent with a higher melanoma detection rate (16% *versus* 6%), with most cases identified before scheduled visits. Conversely, 52·5% of patients discharged via PIFU for high-risk cSCC did not recontact the clinic, equivalent to 1250 fewer outpatient appointments and €211 693·25 over 2 years, while maintaining a recurrence rate of 2·3%^37^. Notably, all recurrences and new primary lesions in this cohort were detected by the patient. Additionally, PIFU implementation in trauma plastic surgery halved missed appointments, reducing did-not-attend rates from 18% to 9% (*P* < 0·001)^26^.

##### Patient and provider perspectives

Patient perspectives on PIFU in melanoma surveillance reflected both enthusiasm and uncertainty. Many patients valued autonomy, earlier lesion detection, and reduced routine visits, with fear of recurrence driving adherence. Participants undertaking skin self-examinations demonstrated greater thoroughness and consistency than those under SFU; however, some expressed a need for in-person training to reinforce self-monitoring confidence^22,24^. Clinicians endorsed PIFU, especially for patients living in rural areas, noting improved access to dermatology services and fewer unnecessary hospital visits. Earlier melanoma detection via patient-led monitoring was highlighted, but clinicians cautioned that suitability favours digitally literate individuals with a low mole burden, whereas older patients or those with a significant number of moles require enhanced support^25^.

#### Summary by specialty: urology

##### PIFU methods

Four studies^15,16,18,32^ evaluated PIFU in urology across a range of conditions, from prostate cancer survivorship to bladder pain syndrome (BPS) and postcatheterization management. Remote prostate-specific antigen (PSA) monitoring, combined with patient-led follow-up and nurse-led virtual clinics, has replaced routine in-person surveillance for prostate cancer, with structured education sessions and a holistic needs assessment incorporated to support patient self-management^15^. In patients who had undergone prostatectomy and those discharged with catheters, PIFU involved self-directed catheter removal with written guidance, and patients were advised to attend outpatient clinics if they had not voided by noon^16^. For BPS, self-administered intravesical hyaluronic acid was integrated with video-based training and expedited specialist reaccess^18^. More broadly, another study^32^ explored PIFU in urology FSA clinics (lower urinary tract symptoms, 57 patients; kidney stones, 12; recurrent urinary tract infections, 9; other urological conditions, 22) by a single consultant; those deemed suitable for PIFU were discharged with the clinic number and advised to call and arrange a follow-up appointment should initial management not suffice.

##### Patient volume

One study^16^ noted that 79% of patients successfully removed their catheters at home, with 92% of these voiding successfully and only 8% requiring recatheterization in clinic. Similarly, another^18^ found that patient-led home catheterization and intravesical hyaluronic acid administration for BPS resulted in fewer mean number of hospital visits (7·1 *versus* 12·0; *P* < 0·001), with 77% of patients reporting symptom improvement. For urology FSA clinics, only 2·0% of patients utilized PIFU and requested follow-up appointments^32^.

##### Patient perspectives

Qualitative findings and surveys suggested that PIFU was generally well received, with a significant reduction in unmet needs, an increase in patient satisfaction, an increase in mental health (measured by means of General Health Questionnaire 12), and a decrease in bowel side-effects (assessed by Expanded Prostate Cancer Index Composite 26) observed primarily at 4-month follow-up^15^. However, this difference was not significant at 8-month follow-up. Most patients valued greater autonomy and streamlined access to care, particularly in self-directed catheter removal, which had satisfaction rates exceeding 82%^16^, with a similar satisfaction rate of 97·0% observed by others^16,32^. However, awareness and utilization barriers were common, with up to 43·0% of patients stating that they were unaware of how to utilize PIFU^32^.

##### Economic value

Remote PSA monitoring for follow-up after prostate cancer treatment was associated with lower healthcare costs (€337 *versus* €381 per patient over 8 months)^15^. Another study^18^ estimated that a decline in clinic visits was associated with €1749 per patient saved in the first year and €3733 thereafter.

## Discussion

This systematic review evaluated PIFU across six surgical specialties, and demonstrated that its effects are highly context-dependent. Across most specialties, PIFU reduced routine outpatient utilization without clear evidence of compromised safety; however, the magnitude of reduction varied by clinical context and underlying pathology. In benign conditions, such as fractures or lower urinary tract symptoms, PIFU primarily functions as an event-driven safety net for self-limiting conditions. In contrast, oncological follow-up requires structured safeguards, including surveillance imaging or biochemical monitoring, to mitigate the risk of clinically occult recurrence. These findings suggest that the risk–benefit balance of PIFU differs fundamentally between benign and malignant disease contexts.

This meta-analysis has illustrated that PIFU utilization is not a uniform construct but varies substantially according to pathway design. The extreme heterogeneity observed (*I*^2^ = 95%) and wide prediction interval indicate that utilization should not be interpreted as a fixed or specialty-level characteristic. Instead, two conceptual models appear to emerge: discharge-based PIFU, in which patients are discharged following resolution of an acute episode and further contact is event-driven, and monitoring-based PIFU, in which ongoing symptom fluctuation is anticipated and re-engagement may represent appropriate disease management rather than pathway failure. Injection-monitoring pathways demonstrated significantly higher utilization^43^; however, this association may reflect differences in underlying chronicity, follow-up duration, and patient selection rather than pathway structure alone. Importantly, utilization rates are value-neutral: lower recontact may reflect effective discharge but could also indicate disengagement, whereas higher utilization may represent either unmet need or appropriate symptom-driven care. These findings underscore that the success of PIFU models is highly context-dependent and should be evaluated in relation to clinical intent rather than absolute utilization thresholds.

Furthermore, a systematic review of PIFU models in cancer found that healthcare professionals expressed concerns regarding the loss of clinician oversight, particularly in higher-risk patient groups^3^. Qualitative evidence from head and neck cancer populations has further demonstrated that deficits in symptom awareness and variable patient compliance can undermine the safety and acceptability of PIFU, especially when early recurrence is subtle clinically. Both patients and clinicians described uncertainty in recognizing concerning symptoms and engaging appropriately with follow-up pathways, disproportionately affecting individuals with lower health literacy, ongoing smoking or alcohol use, or in psychological distress, reinforcing the need for enhanced education and low-threshold rapid reaccess mechanisms in these settings^27,28^. Digital tools, such as electronic PROs and symptom-triggered alerts, can improve patient safety and confidence in self-monitoring of symptoms. Several PIFU models utilized digital solutions to provide asynchronous surveillance and automated red-flag alerts^13,15,20,22,41^. Such platforms not only reduce the manual triage workload but also extend specialist input to geographically isolated patients, mitigating travel costs and time off work barriers^45,46^.

Potential barriers and risks exist for PIFU, notably delayed detection of complications, missed disease recurrence, patient disengagement, cost of implementation, and, in some instances, increased workload. For example, the intervention cohort in the MEL-SELF RCT^22^ experienced a 1·5-fold increase in clinic visits compared with the SFU group, which may reflect earlier detection but also represents a potential increase in downstream workload and patient anxiety. A systematic review^47^ of qualitative studies found that patients are understandably hesitant to rely on self-assessment and may even avoid checking due to recurrence-related anxiety. This risk increases in settings where self-assessment may prove difficult at home, such as in oropharyngeal carcinoma, where most local recurrences are detected at routine follow-up^48^. Similarly, another study^33^ noted that most rectal cancer recurrences were identified through routine imaging rather than patient-initiated contact, reinforcing the need for structured safety nets. Finally, the PIFU system increases the risk of loss to follow-up, raising concerns about medicolegal liability. Litigation commonly arises when abnormal findings are not actioned, underscoring the need for clear delineation of responsibility, robust electronic medical record flagging for missed appointments, and documented shared decision-making as adjuncts to successful PIFU implementation.

PIFU has demonstrated cost savings primarily through reduced outpatient visits, with studies^11,37^ reporting estimated savings of at least €200 000 as a result of fewer clinics. However, the initial implementation costs, including staff training and digital infrastructure, must be considered. A Cochrane review^4^ found mixed evidence on savings, with some studies showing little to no cost difference when factoring in implementation costs or digital infrastructure requirements. Additionally, the economic incentives for PIFU are highly context-specific. In tax-funded public systems, such as Aotearoa New Zealand and the UK, outpatient appointments represent a net cost; reducing them frees resources for FSAs. Conversely, in fee-for-service environments, such as the USA or private healthcare systems, follow-up appointments generate revenue, which PIFU may threaten.

This review has several limitations. First, the heterogeneity of PIFU models, variability in eligibility criteria, and variation in follow-up protocols across specialties limit the generalizability of PIFU. Selection bias was substantial across each specialty, with PIFU being offered preferentially to patients perceived as young, reliable, digitally literate, or at low risk, further limiting generalizability. Confounding by indication was also common, particularly in observational studies, where outcomes may reflect idealistic patient characteristics rather than the PIFU model itself. Moreover, each specialty presents its own unique set of challenges and pathologies which may not be amenable to PIFU principles. Second, a limited number of RCTs was available, with several larger trials currently ongoing, which limited the strength of the data available. Third, there was a scarcity of data evaluating true cost analysis, with many studies hypothesizing that a reduction in clinic volume would lead to a congruent reduction in costs. Finally, all studies included in this review were from high-income countries with access to a readily contactable patient population and digital infrastructure for remote monitoring, limiting their applicability to low- and middle-income countries.

In conclusion, PIFU models show potential to reduce outpatient burden in selected surgical settings; however, their benefits are highly contextual and must be balanced against risks related to patient safety, engagement, and resource allocation. Although the success of PIFU varies by specialty, well designed models may enhance patient autonomy without increasing adverse outcomes. Hybrid approaches that integrate PIFU with fewer scheduled reviews may optimize safety for high-risk patients, particularly in oncology. Future research should focus on implementing PIFU into existing healthcare frameworks to maximize its benefits and acceptability, and evaluate its long-term cost-effectiveness.

## Supplementary Material

zrag147_Supplementary_Data

## Data Availability

The data supporting the findings of this study are available from the corresponding author upon reasonable request.

## References

[1] Sherlaw-Johnson C, Georghiou T, Reed S, Hutchings R, Appleby J, Bagri S et al Investigating innovations in outpatient services: a mixed-methods rapid evaluation. Health Soc Care Deliv Res 2024;12:1–162 10.3310/VGQD4611 39331466

[2] Chan A, Green K, Kontra K, Chapman T, Wewerka C, Statham A. The Royal Berkshire NHS Foundation Trust outpatient services transformation programme to improve quality and effectiveness of patient care. Future Healthc J 2022;9:255–261 10.7861/fhj.2021-0150 PMC9761465 36561832

[3] Chhatwal K, Deighton AJ. Patient-initiated follow up as a means of reducing pressures in secondary care. Intern Med J 2022;52:683–685 10.1111/imj.15741 35419966

[4] Whear R, Thompson-Coon J, Rogers M, Abbott RA, Anderson L, Ukoumunne O et al Patient-initiated appointment systems for adults with chronic conditions in secondary care. Cochrane Database Syst Rev 2020; (4)CD010763 10.1002/14651858.CD010763.pub2 PMC7144896 32271946

[5] Newton C, Beaver K, Clegg A. Patient initiated follow-up in cancer patients: a systematic review. Front Oncol 2022;12:954854 10.3389/fonc.2022.954854 PMC9606321 36313728

[6] Page MJ, McKenzie JE, Bossuyt PM, Boutron I, Hoffmann TC, Mulrow CD et al The PRISMA 2020 statement: an updated guideline for reporting systematic reviews. Int J Surg 2021;88:105906 10.1016/j.ijsu.2021.105906 33789826

[7] Pitesa R, Hill AG. *Patient-Initiated Follow-Up (PIFU) in Surgery: A Systematic Review*. https://www.crd.york.ac.uk/PROSPERO/view/CRD42025648072 (accessed 02 March 2025)

[8] Sterne JAC, Savović J, Page MJ, Elbers RG, Blencowe NS, Boutron I et al Rob 2: a revised tool for assessing risk of bias in randomised trials. BMJ 2019;366:l4898 10.1136/bmj.l4898 31462531

[9] Wells G, Shea B, O’Connell D, Peterson J, Welch V, Losos M et al *The Newcastle-Ottawa Scale (NOS) for Assessing the Quality of Nonrandomised Studies in Meta-Analyses*. https://ohri.ca/sites/ohri/files/2025-09/nosgen.pdf (accessed 30 May 2025)

[10] CASP UK . *CASP Checklist: For Qualitative Research*. https://casp-uk.net/casp-checklists/CASP-checklist-qualitative-2024.pdf (accessed 31 May 2025)

[11] Chadha K, Patrick T, Trowbridge S, Conway A, Waheed S. Open access follow up for breast cancer patients at East Surrey hospital: the way forward for breast cancer care. Eur J Surg Oncol 2014;40:S72

[12] Li E, Rai S, Brown H, Hoar F, Mirza M, Vishwanath L et al P129. If patient initiated follow-up and 5-year mammograms are in place, is routine annual follow-up also necessary for breast cancer patients after completing treatment? Eur J Surg Oncol 2015;41:S63

[13] Batehup L, Porter K, Gage H, Williams P, Simmonds P, Lowson E et al Follow-up after curative treatment for colorectal cancer: longitudinal evaluation of patient initiated follow-up in the first 12 months. Support Care Cancer 2017;25:2063–2073 10.1007/s00520-017-3595-x PMC5445145 28197848

[14] Kirshbaum MN, Dent J, Stephenson J, Topping AE, Allinson V, McCoy M et al Open access follow-up care for early breast cancer: a randomised controlled quality of life analysis. Eur J Cancer Care (Engl) 2017;26:e12577 10.1111/ecc.12577 PMC5516199 27717057

[15] Frankland J, Brodie H, Cooke D, Foster C, Foster R, Gage H et al Follow-up care after treatment for prostate cancer: evaluation of a supported self-management and remote surveillance programme. BMC Cancer 2019;19:368 10.1186/s12885-019-5561-0 PMC6480799 31014282

[16] Hatem E, Tay LJ, Harvey H, Muir G, Brown C. Patient-led trial without catheter (TWOC)—is it feasible? Br J Urol 2019;124:81

[17] Hovdenak Jakobsen I, Juul T, Thaysen HV, Johansen C, Laurberg S. Differences in baseline characteristics and 1-year psychological factors between participants and non-participants in the randomized, controlled trial regarding patient-led follow-up after rectal cancer (FURCA). Acta Oncol 2019;58:627–633 10.1080/0284186X.2019.1581948 30836806

[18] Kitchen MO, Thursby H, Taylor M, Willard K, Mistry-Pain T. Self-administered intravesical hyaluronic acid improves symptoms and quality of life in a patient-centred approach to bladder pain syndrome management. J Endoluminal Endourol 2019;2:e1–e9

[19] Walder E, Cox E. P153: evaluating patient attitudes to patient-led follow-up following breast cancer. Eur J Surg Oncol 2020;46:e51

[20] Riis CL, Stie M, Bechmann T, Jensen PT, Coulter A, Möller S et al ePRO-based individual follow-up care for women treated for early breast cancer: impact on service use and workflows. J Cancer Surviv 2021;15:485–496 10.1007/s11764-020-00942-3 33415653

[21] Jakobsen IH, Thaysen HV, Laurberg S, Johansen C, Juul T. Patient-led follow-up reduces outpatient doctor visits and improves patient satisfaction. One-year analysis of secondary outcomes in the randomised trial Follow-Up after Rectal CAncer (FURCA). Acta Oncol 2021;60:1130–1139 10.1080/0284186X.2021.1950924 34238100

[22] Ackermann DM, Dieng M, Medcalf E, Jenkins MC, van Kemenade CH, Janda M et al Assessing the potential for patient-led surveillance after treatment of localized melanoma (MEL-SELF): a pilot randomized clinical trial. JAMA Dermatol 2022;158:33–42 10.1001/jamadermatol.2021.4704 PMC8771298 34817543

[23] Balhorn J, Su’a B, Jin J, Peng SL, Weston M, Israel L et al Changing the routine: a move to patient initiated follow up to improve surgical outpatient clinic. ANZ J Surg 2022;92:1394–1400 10.1111/ans.17676 PMC9324913 35429226

[24] Drabarek D, Habgood E, Janda M, Hersch J, Ackermann D, Low D et al Experiences of patient-led surveillance, including patient-performed teledermoscopy, in the MEL-SELF pilot randomized controlled trial: qualitative interview study. JMIR Dermatol 2022;5:e35916 10.2196/35916 PMC10334928 37632893

[25] Drabarek D, Habgood E, Ackermann D, Hersch J, Janda M, Morton RL et al Perspectives and experiences of patient-led melanoma surveillance using digital technologies from clinicians involved in the MEL-SELF pilot randomized controlled trial: qualitative interview study. JMIR Dermatol 2022;5:e40623 10.2196/40623 PMC10334935 37632906

[26] Katsura C, Tomouk T, Gathura E, Miranda B, Zweifel C. 305—Improving clinic DNA rates with a patient initiated follow up pathway: a cohort study. Br J Surg 2022;109:znac269.302

[27] Lorenc A, Wells M, Fulton-Lieuw T, Nankivell P, Mehanna H, Jepson M. Clinicians’ views of patient-initiated follow-up in head and neck cancer: a qualitative study to inform the PETNECK2 trial. Clin Oncol 2022;34:230–240 10.1016/j.clon.2021.11.010 PMC8950325 34862101

[28] Lorenc A, Greaves C, Duda J, Brett J, Matheson L, Fulton-Lieuw T et al Exploring the views of patients’ and their family about patient-initiated follow-up in head and neck cancer: a mixed methods study. Eur J Cancer Care (Engl) 2022;31:e13641 10.1111/ecc.13641 PMC9787693 35789510

[29] Moore L, Matheson L, Brett J, Lavender V, Kendall A, Lavery B et al Optimising patient-initiated follow-up care—a qualitative analysis of women with breast cancer in the UK. Eur J Oncol Nurs 2022;60:102183 10.1016/j.ejon.2022.102183 35932754

[30] Stirling PHC, Simpson CJ, Ring D, Duckworth AD, McEachan JE. Virtual management of clinically suspected scaphoid fractures. Bone Joint J 2022;104-B:709–714 10.1302/0301-620X.104B6.BJJ-2021-1464.R2 35638214

[31] Drabarek D, Ackermann D, Medcalf E, Bell KJL. Acceptability of a hypothetical reduction in routinely scheduled clinic visits among patients with history of a localized melanoma (MEL-SELF): pilot randomized clinical trial. JMIR Dermatol 2023;6:e45865 10.2196/45865 PMC10335154 37632976

[32] Hirniak J, Spurr S, Banerjee S. 1038—Introducing a patient-initiated follow up (PIFU) pathway to a district general hospital’s urology department: a two-cycle quality improvement project following the first 100 patients. Br J Surg 2023;110:znad258.535

[33] Hovdenak I, Thaysen HV, Bernstein IT, Christensen P, Hauberg A, Iversen LH et al Quality of life and symptom burden after rectal cancer surgery: a randomised controlled trial comparing patient-led *versus* standard follow-up. J Cancer Surviv 2024;18:1709–1722 10.1007/s11764-023-01410-4 PMC11424718 37395934

[34] Jenkins V, Matthews L, Solis-Trapala I, Gage H, May S, Williams P et al Patients’ experiences of a suppoRted self-manAGeMent pAThway In breast Cancer (PRAGMATIC): quality of life and service use results. Support Care Cancer 2023;31:570 10.1007/s00520-023-08002-z PMC10497681 37698629

[35] Jenkins V, Starkings R, Teoh M, May S, Bloomfield D, Zammit C et al Patients’ views and experiences on the supported self-management/patient-initiated follow up pathway for breast cancer. Support Care Cancer 2023;31:658 10.1007/s00520-023-08115-5 PMC10611591 37889343

[36] Karlsen RV, Høeg BL, Dalton SO, Saltbæk L, Dehlendorff C, Johansen C et al Are education and cohabitation associated with health-related quality of life and self-management during breast cancer follow-up? A longitudinal study. Acta Oncol 2023;62:407–413 10.1080/0284186X.2023.2199128 37083556

[37] Abdul Gafoor SM, Robinson S, Diskantova S, Woodcock E, Yethenpa S, Holloran S et al Patient-initiated follow-up for high-risk cutaneous squamous cell carcinoma: how we do it and 2 years of outcome data. Clin Exp Dermatol 2024;49:1205–1212 10.1093/ced/llae160 38747386

[38] Abed H, Samson D, David M, Abed H, Samson D, David M. Early discharge and patient-initiated follow-up in hand surgery: a new norm following simple hand surgery? Cureus 2024;16:e52493 10.7759/cureus.52493 PMC10874133 38371052

[39] Ackermann D, Hersch J, Jordan D, Clinton-Gray E, Bracken K, Janda M et al Participant motivators and expectations in the MEL-SELF randomized clinical trial of patient-led surveillance for recurrent melanoma: content analysis of survey responses. JMIR Dermatol 2024;7:e58136 10.2196/58136 PMC11528161 39418647

[40] Chaturvedi A, Russell H, Farrugia M, Roger M, Putti A, Jenkins PJ et al Patient-directed follow-up for the clinical scaphoid fracture. Bone Jt Open 2024;5:117–122 10.1302/2633-1462.52.BJO-2023-0119.R1 PMC10853021 38330993

[41] Swartjes H, Aarts CJH, Deuning-Smit E, Vromen HAB, de Wilt JHWH, Vos JAM et al Patient experiences with patient-led, home-based follow-up after curative treatment for colorectal cancer: a qualitative study. BMJ Open 2024;14:e081655 10.1136/bmjopen-2023-081655 PMC10875483 38367967

[42] Younis Z, Hamid MA, Khan MM, Sapra R, Gurukiran G, Singh R. Evaluation of patient-initiated follow-up (PIFU) service in a fracture clinic: a comprehensive service evaluation and patient satisfaction audit. Cureus 2024;16:e73461 10.7759/cureus.73461 PMC11633848 39664163

[43] Fan KS, Haq I, Taha A, Carne A, Gademsetty C, Solan M. Efficient patient-initiated follow-up after orthopaedic injections. Eur J Orthop Surg Traumatol 2025;35:77 10.1007/s00590-025-04193-9 PMC11861138 39998697

[44] Naskar R, Garg V, Middleton E, Moverley R. The impact and utilisation of patient-initiated follow-ups (PIFU) in the elective upper-limb clinic at secondary care. Ann R Coll Surg Engl 2025:1–5. DOI:10.1308/rcsann.2025.0073 [Epub ahead of print] PMC13529383 41117163

[45] Hunter I, Lockhart C, Rao V, Tootell B, Wong S. Enabling rural telehealth for older adults in underserved rural communities: focus group study. JMIR Form Res 2022;6:e35864 10.2196/35864 PMC9675008 36331533

[46] Mancini R, Bartolo M, Pattaro G, Ioni L, Picconi T, Pernazza G. The role of telemedicine in the postoperative home monitoring after robotic colo-rectal cancer surgery: a preliminary single center experience. Updates Surg 2022;74:171–178 10.1007/s13304-021-01132-1 34313956

[47] Dretzke J, Lorenc A, Adriano A, Herd C, Mehanna H, Nankivell P et al Systematic review of patients’ and healthcare professionals’ views on patient-initiated follow-up in treated cancer patients. Cancer Med 2023;12:16531–16547 10.1002/cam4.6243 PMC10469665 38771977

[48] Landström F, Reizenstein J, Kristiansson S. Detection of recurrence after primary treatment for oropharyngeal carcinoma. Anticancer Res 2022;42:5597–5600 10.21873/anticanres.16067 36288847

