# Supplementary material for: Patient-initiated follow-up in surgical specialties: meta-analysis and meta-regression
Source: BJS Open. 2026 Sep 21;10(5):zrag147. doi: 10.1093/bjsopen/zrag147 (PMC13591271; doi:10.1093/bjsopen/zrag147)
Supplement: Supplementary file 1 [file zrag147_supplementary_data.docx]

**Patient Initiated Follow Up in Surgical Specialities: A Systematic Review, Meta-Analysis and Meta-Regression**

Renato Pitesa^1^ (MBChB), Stephanie Moody^2^ (MBChB), Andrew G. Hill^1,2^ (MBChB, FRACS, FRSNZ)

^1^Department of Surgery, The University of Auckland, Auckland, New Zealand

^2^Department of General Surgery, Middlemore Hospital, Auckland, New Zealand

**Corresponding author.** Renato Pitesa

Level 2, North Wing, Esmé Green Building 30, Middlemore Hospital, 100 Hospital Road, Otahuhu, Auckland, 2025, New Zealand

**orcid.org/0000-0002-8004-6716**; **@RenatoPitesa**

**Supplementary Materials - Index**

| **Supplementary Methods** |  |
| --- | --- |
| PRISMA Checklist | *pag. 2* |
| PRISMA Abstract Checklist | *pag. 6* |
| Search Strategy According to Database | *pag. 7* |
| **Supplementary Tables** |  |
| Characteristics of Included Studies | *pag. 8* |
| Newcastle-Ottowa Scale Quality Assessment of Non-Randomised Studies | *pag. 20* |
| CASP Quality Assessment of Qualitative Studies | *pag. 22* |
| **References** | *pag. 30* |
|  |  |

**Supplementary Methods: PRISMA Checklist**

| **Topic** | **No.** | **Item** | **Location where item is reported** |
| --- | --- | --- | --- |
| **TITLE** |  |  |  |
| **Title** | 1 | Identify the report as a systematic review. | page 1 |
| **ABSTRACT** |  |  |  |
| **Abstract** | 2 | See the PRISMA 2020 for Abstracts checklist |  |
| **INTRODUCTION** |  |  |  |
| **Rationale** | 3 | Describe the rationale for the review in the context of existing knowledge. | page 4 |
| **Objectives** | 4 | Provide an explicit statement of the objective(s) or question(s) the review addresses. | page 4 |
| **METHODS** |  |  |  |
| **Eligibility criteria** | 5 | Specify the inclusion and exclusion criteria for the review and how studies were grouped for the syntheses. | page 6 |
| **Information sources** | 6 | Specify all databases, registers, websites, organisations, reference lists and other sources searched or consulted to identify studies. Specify the date when each source was last searched or consulted. | page 5 |
| **Search strategy** | 7 | Present the full search strategies for all databases, registers and websites, including any filters and limits used. | supplementary methods 3 |
| **Selection process** | 8 | Specify the methods used to decide whether a study met the inclusion criteria of the review, including how many reviewers screened each record and each report retrieved, whether they worked independently, and if applicable, details of automation tools used in the process. | page 6-7 |
| **Data collection process** | 9 | Specify the methods used to collect data from reports, including how many reviewers collected data from each report, whether they worked independently, any processes for obtaining or confirming data from study investigators, and if applicable, details of automation tools used in the process. | page 7 |
| **Data items** | 10a | List and define all outcomes for which data were sought. Specify whether all results that were compatible with each outcome domain in each study were sought (e.g. for all measures, time points, analyses), and if not, the methods used to decide which results to collect. | page 7 |
|  | 10b | List and define all other variables for which data were sought (e.g. participant and intervention characteristics, funding sources). Describe any assumptions made about any missing or unclear information. | page 7 |
| **Study risk of bias assessment** | 11 | Specify the methods used to assess risk of bias in the included studies, including details of the tool(s) used, how many reviewers assessed each study and whether they worked independently, and if applicable, details of automation tools used in the process. | page 7-8 |
| **Effect measures** | 12 | Specify for each outcome the effect measure(s) (e.g. risk ratio, mean difference) used in the synthesis or presentation of results. | page 8-9 |
| **Synthesis methods** | 13a | Describe the processes used to decide which studies were eligible for each synthesis (e.g. tabulating the study intervention characteristics and comparing against the planned groups for each synthesis (item 5)). | page 8-9 |
|  | 13b | Describe any methods required to prepare the data for presentation or synthesis, such as handling of missing summary statistics, or data conversions. | page 8-9 |
|  | 13c | Describe any methods used to tabulate or visually display results of individual studies and syntheses. | page 8-9 |
|  | 13d | Describe any methods used to synthesize results and provide a rationale for the choice(s). If meta-analysis was performed, describe the model(s), method(s) to identify the presence and extent of statistical heterogeneity, and software package(s) used. | page 8-9 |
|  | 13e | Describe any methods used to explore possible causes of heterogeneity among study results (e.g. subgroup analysis, meta-regression). | page 8-9 |
|  | 13f | Describe any sensitivity analyses conducted to assess robustness of the synthesized results. | page 8-9 |
| **Reporting bias assessment** | 14 | Describe any methods used to assess risk of bias due to missing results in a synthesis (arising from reporting biases). | page 8-9 |
| **Certainty assessment** | 15 | Describe any methods used to assess certainty (or confidence) in the body of evidence for an outcome. | NA |
| **RESULTS** |  |  |  |
| **Study selection** | 16a | Describe the results of the search and selection process, from the number of records identified in the search to the number of studies included in the review, ideally using a flow diagram. | Figure 1 |
|  | 16b | Cite studies that might appear to meet the inclusion criteria, but which were excluded, and explain why they were excluded. | Figure 1 |
| **Study characteristics** | 17 | Cite each included study and present its characteristics. | Table 1; supplementary table 1 |
| **Risk of bias in studies** | 18 | Present assessments of risk of bias for each included study. | Figure 2-3; supplementary table 2-3 |
| **Results of individual studies** | 19 | For all outcomes, present, for each study: (a) summary statistics for each group (where appropriate) and (b) an effect estimate and its precision (e.g. confidence/credible interval), ideally using structured tables or plots. | Page 6-15 |
| **Results of syntheses** | 20a | For each synthesis, briefly summarise the characteristics and risk of bias among contributing studies. | page 9-10 |
|  | 20b | Present results of all statistical syntheses conducted. If meta-analysis was done, present for each the summary estimate and its precision (e.g. confidence/credible interval) and measures of statistical heterogeneity. If comparing groups, describe the direction of the effect. | Figure 4; page 16 |
|  | 20c | Present results of all investigations of possible causes of heterogeneity among study results. | Page 16-17 |
|  | 20d | Present results of all sensitivity analyses conducted to assess the robustness of the synthesized results. | figure 4 |
| **Reporting biases** | 21 | Present assessments of risk of bias due to missing results (arising from reporting biases) for each synthesis assessed. | NA |
| **Certainty of evidence** | 22 | Present assessments of certainty (or confidence) in the body of evidence for each outcome assessed. | NA |
| **DISCUSSION** |  |  |  |
| **Discussion** | 23a | Provide a general interpretation of the results in the context of other evidence. | page 21-23 |
|  | 23b | Discuss any limitations of the evidence included in the review. | page 23-24 |
|  | 23c | Discuss any limitations of the review processes used. | page 23-24 |
|  | 23d | Discuss implications of the results for practice, policy, and future research. | page 24 |
| **OTHER INFORMATION** |  |  |  |
| **Registration and protocol** | 24a | Provide registration information for the review, including register name and registration number, or state that the review was not registered. | page 5 |
|  | 24b | Indicate where the review protocol can be accessed, or state that a protocol was not prepared. | page 5 |
|  | 24c | Describe and explain any amendments to information provided at registration or in the protocol. | NA |
| **Support** | 25 | Describe sources of financial or non-financial support for the review, and the role of the funders or sponsors in the review. | page 25 |
| **Competing interests** | 26 | Declare any competing interests of review authors. | page 25 |
| **Availability of data, code and other materials** | 27 | Report which of the following are publicly available and where they can be found: template data collection forms; data extracted from included studies; data used for all analyses; analytic code; any other materials used in the review. | page 25 |

**Supplementary Methods: PRISMA Abstract Checklist**

| **Topic** | **No.** | **Item** | **Reported?** |
| --- | --- | --- | --- |
| **TITLE** |  |  |  |
| **Title** | 1 | Identify the report as a systematic review. | Yes |
| **BACKGROUND** |  |  |  |
| **Objectives** | 2 | Provide an explicit statement of the main objective(s) or question(s) the review addresses. | Yes |
| **METHODS** |  |  |  |
| **Eligibility criteria** | 3 | Specify the inclusion and exclusion criteria for the review. | No |
| **Information sources** | 4 | Specify the information sources (e.g. databases, registers) used to identify studies and the date when each was last searched. | Yes |
| **Risk of bias** | 5 | Specify the methods used to assess risk of bias in the included studies. | Yes |
| **Synthesis of results** | 6 | Specify the methods used to present and synthesize results. | Yes |
| **RESULTS** |  |  |  |
| **Included studies** | 7 | Give the total number of included studies and participants and summarise relevant characteristics of studies. | Yes |
| **Synthesis of results** | 8 | Present results for main outcomes, preferably indicating the number of included studies and participants for each. If meta-analysis was done, report the summary estimate and confidence/credible interval. If comparing groups, indicate the direction of the effect (i.e. which group is favoured). | Yes |
| **DISCUSSION** |  |  |  |
| **Limitations of evidence** | 9 | Provide a brief summary of the limitations of the evidence included in the review (e.g. study risk of bias, inconsistency and imprecision). | No |
| **Interpretation** | 10 | Provide a general interpretation of the results and important implications. | Yes |
| **OTHER** |  |  |  |
| **Funding** | 11 | Specify the primary source of funding for the review. | No |
| **Registration** | 12 | Provide the register name and registration number. | Yes |

**Supplementary Methods: Search Strategy According to Database**

**Database: Ovid Embase**

1	(patient-led or patient led or patient-initiated or patient initiated or PIFU).mp.

2	exp "Appointments and Schedules"/ or (follow-up or follow up).mp. or (appoint* or book* or schedul*).ti.

3	outpatient.mp. or exp Outpatients/ or exp Ambulatory Care Facilities/ or clinic.ti.

4	1 and 2 and 3

5	1 and 2

6	1 and 3

7	5 or 6

8	limit 7 to (english language and yr="2014 -Current")

**Database: Ovid MEDLINE(R) ALL**
1  (patient-led or patient led or patient-initiated or patient initiated or PIFU).mp.
2  exp "Appointments and Schedules"/ or (follow-up or follow up).mp. or (appoint* or book* or schedul*).ti.
3  outpatient.mp. or exp Outpatients/ or exp Ambulatory Care Facilities/ or clinic.ti.
4  1 and 2 and 3
5  1 and 2
6  1 and 3
7  5 or 6
8  limit 7 to (english language and yr="2014 -Current")

**Database: CENTRAL**

#1	"patient led":ti,ab

#2	"patient initiated":ti,ab

#3	PIFU

#4	MeSH descriptor: [Aftercare] explode all trees

#5	MeSH descriptor: [Appointments and Schedules] explode all trees

#6	"follow up":ti,ab

#7	appointment*:ti,ab

#8	book*:ti,ab

#9	schedul*:ti,ab

#10	outpatient:ti,ab

#11	MeSH descriptor: [Ambulatory Care Facilities] explode all trees

#12	MeSH descriptor: [Outpatients] explode all trees

#13	clinic*:ti,ab

#14	outpatient*:ti,ab

#15	#1 OR #2 OR #3

#16	#6 OR #7 OR #8 OR #9 OR #10

#17	#11 OR #12 OR #13 OR #14

#18	#15 AND #16 AND #17

**Supplementary Table S1: Characteristics of Included Studies**

| **Citation** | **Specialty** | **Study design** | **Setting** | **Eligibility criteria** | **Intervention** | **Comparator** | **Outcomes** | **Timeline/Sample size** |
| --- | --- | --- | --- | --- | --- | --- | --- | --- |
| Chadha et al, 2014 (1) | Breast (malignant) | Retrospective cohort | UK | Breast cancer (BC) survivors transitioning to follow up care | PIFU | SFU | Clinic volume  Patient safety  Cost-analysis | 100 clinic appointments analysed |
| Li et al, 2015 (2) | Breast (malignant) | Retrospective cohort | UK | BC survivors with 3 years of follow up | PIFU + annual mammograms for 5 years | SFU | Recurrence detection rates across: Mammography  Patient-initiated symptom detection  Clinical follow-up detection | 590 participants |
| Batehup et al, 2017 (3) | Colorectal (malignant) | Qualitative | UK | Completion of all treatment with curative intent for CRC; absence of a second cancer diagnosed  during treatment; age 18 years or over; no dementia, severe  memory loss, or learning disability preventing questionnaire  completion. | PIFU + self-management workshop + remote surveillance | SFU | QoL  PIFU acceptability  Cost-analysis | 251 participants  SFU (pre-PIFU implementation): 75 participants  SFU (post-PIFU implementation): 92 participants  PIFU: 84 participants |
| Kirshbaum et al, 2017 (4) | Breast (malignant) | Randomised Controlled Trial (RCT) | UK | Stage 1 or 2 BC survivors and considered to be clinically at low risk of recurrence | PIFU + resource/education pack (n = 56) | SAU (n = 56) | Impact of open-access PIFU on health-related quality of life (HRQoL).  Patient anxiety and depression (HADS).  Functional well-being (EORTC QLQ-C30, QLQ-BR23).  Patient satisfaction and confidence in follow-up care. | 112 participants |
| Frankland et al, 2019 (5) | Urology (malignant) | Comparative cohort | UK | Prostate cancer (PC) survivors within three years of completion of RP or RT, or within  three years of commencement of PADT | PIFU + remote PSA surveillance + self-management workshop + Holistic needs assessment | SFU | Unmet survivorship needs (Cancer Survivors’ Unmet Needs Survey, CaSUN).  Cancer-specific quality of life (FACT-G).  Psychological well-being (GHQ-12).  Fear of recurrence (Worry of Cancer Scale).  Bowel, urinary, and sexual symptoms (EPIC-26).  Patient satisfaction with follow-up care.  Health economics: Cost-effectiveness compared to standard care. | 627 participants PIFU: 293  SFU: 334 |
| Hatem et al, 2019 (6) | Urology (benign) | Prospective cohort | UK | Patients undergoing laser prostatectomy or other procedures requiring post-operative catheterization. | PIFU + instructions on how to remove catheter safely at home + safety net instructions to visit clinic if unable to void by noon | SFU | Feasibility and success rate of patient-led TWOC.  Complications requiring hospital attendance.  Patient satisfaction with the process. | 62 participants |
| Jakobsen et al, 2019 (7) | Colorectal (malignant) | RCT | Denmark | Patients with rectal cancer (Stage < IV) who had undergone curative surgery | (FURCA) PIFU + routine CT scans at 1 and 3 years postop | SFU 6- and 12-month appointments | QoL and symptom burden  Total number of hospital contacts Number of outpatient doctor visits Patient-reported involvement and satisfaction | 149 (Participants)  113 (non-participants) |
| Kitchen et al, 2019 (8) | Urology (benign) | Prospective cohort | UK | Patients diagnosed with bladder pain syndrome (BPS) undergoing HA treatment. | PIFU + written and video-based instructional support on intermittent self-catheterisation training | SFU | Patient-reported symptom improvement (O’Leary-Sant Interstitial Cystitis Symptom Index, ICSI).  Patient volume and costs | 60 participants Recurrent UTIs – 29 patients  Pelvic/bladder pain – 24 patients  Refractory lower urinary tract symptoms (LUTS) – 7 patients |
| Walder & Cox, 2020 (9) | Breast (malignant) | Qualitative | UK | Breast cancer survivors | PIFU | SFU | Patient confidence in self-managing follow-up | 69 responses analysed |
| Riis et al, 2021 (10) | Breast (malignant) | Mixed-methods RCT | Denmark | Women, age > 50, who were postmenopausal at the time of diagnosis, had histologically verified hormone-receptor-positive BC, underwent primary surgery with curative intent, and were scheduled to undergo 5 years of adjuvant endocrine therapy | PIFU + ePRO + mammogram every 18-24 months | SFU | Impact on outpatient service use, workload and workflow  PROs | 134 participants (207 eligible) |
| Jakobsen et al, 2021 (11) | Colorectal (malignant) | RCT 1-year analysis | Denmark | Patients with rectal cancer (Stage < IV) who had undergone curative surgery | FURCA | SFU 6- and 12-month appointments | QoL and symptom burden  Total number of hospital contacts Number of outpatient doctor visits Patient-reported involvement and satisfaction | 366 participants |
| Ackermann et al, 2022 (12) | Plastics (malignant) | Mixed-methods Pilot RCT | Australia | Patients previously treated for localized melanoma (Stage 0–II) | (MEL-SELF) PIFU + Total body skin self-examination (SSE) with  dermatoscope attachment for smartphones to capture teledermoscopy images +  Images reviewed by a teledermatologist within 3 working days +  Regular reminders via email/SMS to perform SSE every 2 months. | SFU | Adherence to teledermoscopy. Usability of digital tools. Factors influencing engagement. Psychological impact and fear of recurrence. | 100 participants  PIFU: 49  SFU: 51 |
| Balhorn et al, 2022 (13) | Colorectal (benign) | Retrospective and prospective cohort | New Zealand | Patients referred with rectal bleeding | PIFU | SFU | Safety and feasibility of PIFU in reducing unnecessary follow-up appointments without increasing missed cancer diagnoses  PIFU utilisation ED presentation Elective surgery rates Investigations performed 5-year follow-up | 268 participants  133 (SFU), 135 (PIFU) |
| Drabarek et al, 2022 (14) | Plastics (malignant) | Qualitative | Australia | Patients randomised to the intervention arm (patient-led surveillance) of the MEL-SELF pilot RCT | MEL-SELF | SFU | Patient experiences and perceptions of patient-led surveillance. | 49 eligible patients invited 43 responded (88%) 20 agreed to participate in the interviews |
| Drabarek et al, 2022 (15) | Plastics (malignant) | Qualitative | Australia | Clinicians involved in the MEL-SELF pilot RCT | MEL-SELF | SFU | Clinician experiences and perspectives on the use of patient-led surveillance with digital tools. | 8 clinicians interviewed (out of 11 invited). 4 dermatologists (3 teledermatologists, 2 treating clinicians). 1 surgical oncologist. 3 general practitioners (GPs) with skin cancer screening expertise. |
| Katsura et al, 2022 (16) | Plastics (benign) | Prospective cohort | UK | Patients requiring plastic surgery trauma outpatient follow-up. | PIFU within 3 months of injury | SFU | Change in clinic DNA rates.  Identification of specific patient groups most likely to DNA. | 823 patients (pre-PIFU, 2018) 879 patients (post-PIFU, 2021) |
| Lorenc et al, 2022 (17) | ORL (malignant) | Qualitative | UK | Head and neck clinicians (HNCs) | (PETNECK2) PIFU + Negative 12-month PET scan | SFU | Clinicians' perceptions of PIFU and barriers/facilitators to its implementation. | 34 HNC clinicians: 10 oncologists 10 surgeons (ENT and maxillofacial) 8 clinical nurse specialists (CNSs) 4 speech and language therapists (SLTs) 1 dietitian 1 radiographer Representing 13 different HNC centres across England, Wales, and Scotland. |
| Lorenc et al, 2022 (18) | ORL (malignant) | Qualitative | UK | HNC patients (≤3 years post-treatment) | PETNECK2 | SFU | Patients’ perceptions of current follow up and PIFU and barriers/facilitators to its implementation. | 144 patients completed the survey 30 patients participated in interviews |
| Moore et al, 2022 (19) | Breast (malignant) | Qualitative | UK | BC survivors | PIFU | SFU | Patient experiences with PIFU Barriers and facilitators to seeking help Emotional well-being and fear of recurrence | 20 participants (from 118) |
| Stirling et al, 2022 (20) | Orthopaedic (benign) | Prospective cohort | UK | Patients with a suspected scaphoid fracture not visible on initial X-ray. | PIFU + wrist splint + self-monitoring checklist | SFU | Number of patients requiring follow-up or re-evaluation. | 221 participants |
| Drabarek et al, 2023 (21) | Plastics (malignant) | Qualitative | Australia | Patients previously treated for localised melanoma (Stages 0–II) | MEL-SELF | SFU | Acceptability of a hypothetical reduction in routinely scheduled visits. | 87 answered the questionnaire at baseline  66 answered the questionnaire at 6 months  79 provided a free-text explanation at either time point |
| Hirniak et al, 2023 (22) | Urology (benign) | Retrospective cohort | UK | New patients seen under a single consultant in urology FSA clinics | PIFU | SFU | Utilisation of PIFU and patient satisfaction  Awareness and accessibility of the PIFU pathway. Effectiveness in reducing unnecessary outpatient appointments. | First 100 patients evaluated over an average of 6 months on PIFU.  Lower urinary tract symptoms (LUTS) n=57  Kidney stones n=12  Recurrent urinary tract infections (UTIs) n=9  Other urological conditions n=22 |
| Hovdenak et al, 2023 (23) | Colorectal (malignant) | RCT | Denmark | Patients with rectal cancer (Stage < IV) who had undergone curative surgery | FURCA | SFU—Five OP visits over 3 years | Health-related quality of life (HRQoL) and symptom burden measured using the Functional Assessment of Cancer Therapy - Colorectal (FACT-C) score  Functional outcomes Patient involvement and satisfaction Cancer recurrence rates at 3 years Number and types of follow-up contacts | 248 completed the full three-year follow-up (26% dropout rate). |
| Jenkins et al 2023 (24) | Breast (malignant) | Prospective cohort | UK | Early-stage BC survivors transitioning to follow up | PIFU | SFU—six monthly for 5 years | Quality of life (QoL) trends measured using FACT-B, EQ-5D-5L Psychological morbidity (GHQ-12) Self-efficacy (GSE) Service utilisation (frequency of contact with hospital services) | 110 participants (78% recruitment rate) |
| Jenkins et al, 2023 (25) | Breast (malignant) | Qualitative | UK | Early-stage BC survivors transitioning to follow up | PIFU | SFU: 6 monthly for 5 years | Patient experiences and confidence in managing follow-up through PIFU. | 32 participants (from 110 sample) |
| Karlsen et al, 2023 (26) | Breast (malignant) | Prospective cohort | Denmark | Patients with low or intermediate risk of recurrence (> 40 years of age at diagnosis, stage I-II, without a BRCA gene mutation, and not having had recurrent BC).  Patients 1-5 years after BC surgery could choose between PIFU or SFU ever 6-12 months  Patients >5 years after BC surgery were solely offered PIFU | PIFU | SFU: 6- to 12- monthly | Health-related quality of life (HRQoL) using QLQ-C30 and QLQ-BR23  Confidence in self-management and recognition of symptoms | 987 participants (1773 invited) |
| Abdul Gafoor et al, 2024 (27) | Plastics (malignant) | Prospective cohort | UK | Patients with high-risk cutaneous squamous cell carcinoma (cSCC), as defined by British Association of Dermatologists (BAD) guidelines. | PIFU + Verbal and written education on self-monitoring for recurrence.  + A letter outlining “red flag” symptoms and self-examination techniques. | SFU | Safety and feasibility of PIFU in reducing unnecessary follow-up appointments without increasing missed recurrences or metastases  Clinic volume Associated cost savings. | 417 patients included in the final analysis (59 excluded) |
| Abed et al, 2024 (28) | Orthopaedic (benign) | Prospective Cohort | UK | Patients undergoing simple hand surgery under local anaesthesia. | PIFU + Postoperative instructions + hand exercises | SFU | Number of patients requiring follow-up after early discharge.  Reasons for follow-up requests. Complications and safety. Patient satisfaction | 105 participants Carpal tunnel decompression (n=83) Trigger finger release (n=9) Steroid injection into a joint (n=13) |
| Ackermann et al, 2024 (29) | Plastics (malignant) | Qualitative | Australia | Patients previously treated for localised melanoma (Stage 0–II), enrolled in the MEL-SELF trial | MEL-SELF | SFU | Participant motivations and expectations for engaging in a PIFU-based melanoma trial.  Themes influencing trial recruitment, retention, and adherence. Engagement Barriers and facilitators to participation. | 100 participants analysed (first 100 randomised in the trial) 98 provided responses about motivations 97 provided responses about expectations |
| Chaturvedi et al, 2024 (30) | Orthopaedic (benign) | Prospective cohort | UK | Patients with a suspected clinical scaphoid fracture | PIFU + wrist splint + verbal and written information | SFU | Number of patients requiring follow-up or re-evaluation.  Missed complications  Cost savings | 442 participants |
| Swartjes et al, 2024 (31) | Colorectal (malignant) | Qualitative | Netherlands | CRC patients post curative treatment who were disease-free at 12 months post-resection and competent in using digital health tools. | PIFU + remote CEA surveillance | SFU | Patient experiences with PIFU | 12 patients |
| Younis et al, 2024 (32) | Orthopaedic (benign) | Retrospective cohort | UK | Orthopaedic patients discharged from fracture clinic | PIFU | NA | Patient satisfaction, service use | 204 patients |
| Fan et al, 2025 (33) | Orthopaedic (benign) | Prospective cohort | UK | Orthopaedic patients discharged from elective foot and ankle clinic | PIFU | NA | Patient engagement and patient re-presentation | 104 patients |
| Naskar et al, 2025 (34) | Orthopaedic (benign) | Retrospective cohort | UK | Orthopaedic patients discharged from elective upper limb (shoulder and elbow) clinic | PIFU | NA | Patient engagement and patient re-presentation | 353 consultations, 81 PIFU patients |
| PRAGMATIC — **P**atients' experiences of a suppo**R**ted self-man**AG**e**M**ent p**AT**hway **I**n breast **C**ancer  FURCA — **F**ollow-**U**p after **R**ectal **CA**ncer  PETNECK2 — **PET**-CT guided, symptom-based, patient-initiated surveillance versus clinical follow-up in head **NECK** cancer  MEL-SELF —**MEL**anoma **SELF-**surveillencve | | | | | | | | |

**Supplementary Table S2: Newcastle-Ottowa Scale Quality Assessment of Non-Randomised Studies**

| **Study** | **Selection (4)** | | | | **Comparability (2)** | **Outcome (3)** | | | **Total (9)** |
| --- | --- | --- | --- | --- | --- | --- | --- | --- | --- |
|  | **Representativeness of the exposed cohort** | **Selection of the non-exposed cohort** | **Ascertainment of exposure** | **Demonstration that outcome of interest was not present at start of study** | **Comparability of cohorts on the basis of the design or analysis** | **Assessment of outcome** | **Was follow-up long enough for outcomes to occur** | **Adequacy of follow up of cohorts** |  |
| Batehup 2017 (3) | A* | A* | A* | A* | * | A* | A* | C | 7/9 |
| Frankland 2019 (5) | A* | A* | A* | A* | ** | A* | A* | B* | 9/9 |
| Balhorn 2022 (13) | A* | A* | A* | A* | * | A* | A* | A* | 7/9 |
| Stirling 2022 (20) | A* | - | A* | A* | - | A* | A* | B* | 6/9 |
| Jenkins 2023 (24) | A* | A* | A* | A* | ** | A* | A* | B* | 9/9 |
| Karlsen 2023 (26) | A* | A* | A* | A* | * | A* | A* | B* | 8/9 |
| Abdul Gafoor 2024 (27) | A* | - | A* | A* | - | A* | A* | B* | 6/9 |
| Abed  2024 (28) | A* | - | A* | A* | - | A* | A* | B* | 6/9 |
| Chaturvedi 2024 (30) | A* | - | A* | A* | - | A* | A* | B* | 6/9 |
| Younis 2024 (32) | A* | - | A* | A* | - | A* | A* | B* | 6/9 |
| Fan 2025 (33) | A* | - | A* | A* | - | A* | A* | B* | 6/9 |
| Naskar 2025 (34) | A* | - | A* | A* | - | A* | A* | B* | 6/9 |

**Supplementary Table S3: CASP Quality Assessment of Qualitative Studies**

| **Study** | **Was there a clear statement of the aims of**  **the research?** | **Is a qualitative methodology appropriate?** | **Was the research design appropriate to**  **address the aims of the research?** | **Was the recruitment strategy appropriate to**  **the aims of the research?** | **Was the data collected in a way that**  **addressed the research issue?** | **Has the relationship between researcher and**  **participants been adequately considered?** | **Have ethical issues been taken into**  **consideration?** | **Was the data analysis sufficiently rigorous?** | **Is there a clear statement of findings?** | **How valuable is the research?** |
| --- | --- | --- | --- | --- | --- | --- | --- | --- | --- | --- |
| Riis 2021 (10) | **Yes**:  Mixed-methods study. RCT evaluating impact on patient reported outcomes and qualitative evaluating impact on workload/workflow as per nursing staff involved  *“…to evaluate how individualized follow-up would impact on the workload [and] workflow”.* | **Yes**: Exploratory questions about nurse-navigator experiences warrants an interpretive design; focus-group interview justified as *“well-suited to describe experiences, patterns, and common practice within a profession”* | **Yes/Partial**:  Single 30-min focus group provides only a snapshot; pragmatic but limits depth and group dynamics acknowledged by authors *(“preferably 6–10 participants… smaller groups are easily dominated”).* | **Yes**:  Purposeful sampling of the nurse-navigators who were integral to the pathway; rationale clear (they would *“be the first to recognise the effects”* of PIFU). | **Yes**:  Semi-structured guide, audio-recorded, verbatim transcription; interviewer a specialist nurse not involved in patient care; PI observed. | **Yes**:  Interviewer’s oncology background stated, and potential pre-conception acknowledged *(“preconception… may have influenced interpretation”)* | **Yes**:  Protocol approved by Danish Data Protection Agency and study registered with clinical trials. Informed consent from nurses reported; Danish regulations judged formal REC review unnecessary | **Yes**: Thematic analysis; two analysts iterated between whole-text and coding stages; consensus meetings described | **Yes**: Themes and suggestions for triage automation are presented with illustrative dialogue excerpts | **Yes**: Practical implications for implementing ePRO-PIFU (need for algorithm-driven booking; workload re-allocation). Study limitations discussed |
| Drabarek 2022 (14) | **Yes**:  *“to explore patients’ experiences and perceptions of patient-led surveillance during the 6 months of participation in the MEL-SELF pilot randomized controlled trial.”* | **Yes**:  Semi-structured interviews with thematic analysis to gather subjective experiences | **Yes**:  Serial telephone interviews analysed with an inductive-plus-deductive framework (technology-acceptance model) align well with the exploratory question. | **Yes**:  All 49 intervention-arm participants were invited: 20 consented | **Yes**:  Interviews (13–36 min) were audio-recorded, verbatim-transcribed, and guided by a piloted topic guide; non-verbal notes taken. | **Yes/Can't tell**: Interviewers’ training and non-clinical roles are reported, but discussion of how their positionality might have shaped data collection and interpretation is limited. | **Yes**:  University of Sydney HREC approval and informed consent procedures are detailed. | **Yes**:  Two researchers independently coded transcripts, resolved discrepancies by consensus, and used framework matrices | **Yes**:  Themes are clearly presented. | **Yes**:  Findings inform refinements for the full RCT and highlight broader implementation issues. |
| Drabarek 2022 (15) | **Yes**:  *“…to explore perceptions of potential benefits and harms of mobile teledermoscopy, as well as experiences with this technology, among clinicians participating in a pilot randomized controlled trial of patient-led melanoma surveillance.”* | **Yes**:  A qualitative study nested within the pilot RCT used semi-structured interviews and inductive thematic analysis | **Yes**:  Reflexive thematic analysis of online/phone focus-group–style interviews with dermatologists, skin-specialist GPs and a surgical oncologist aligns with the aims | **Yes**:  Purposive recruitment invited all 11 eligible clinicians; 8 (4 dermatologists, 3 GPs, 1 surgeon) consented, capturing variation in role and geography. | **Yes**:  Interviews (19–77 min) were audio-recorded, verbatim-transcribed and guided by a piloted topic guide; non-verbal notes were taken. | **Yes**:  Interviewer training and independence from participants are stated | **Yes**:  University of Sydney HREC approval and informed consent procedures are detailed. | **Yes**:  Two researchers independently coded transcripts, resolved discrepancies by consensus, and used framework matrices | **Yes**:  Six themes are clearly presented. | **Yes**:  Findings generate actionable strategies for high-value implementation and inform the larger MEL-SELF trial. |
| Lorenc 2022 (17) | **Yes**:  *“This article reports UK clinicians’ views about HNC follow-up and PIFU, to inform the trial design.”* | **Yes**:  Exploratory questions on clinicians’ experiences justifies a qualitative design via online focus groups and inductive thematic analysis | **Yes**:  Focus-group interviews with surgeons, oncologists, nurse specialists and allied health professionals plus thematic analysis closely match aims | **Yes**:  Purposive recruitment via professional bodies/mailing lists across 13 centres captured diverse roles and regions | **Yes**:  Eight Microsoft Teams sessions were audio-recorded, verbatim-transcribed, and guided by a semi-structured topic list; non-verbal cues noted by observers | **Yes**:  Facilitators’ oncology backgrounds and lack of prior relationship are reported | **Yes**:  Ethical approval from the North East Tyne & Wear South REC and HRA (ref 20/NE/0102); confidentiality safeguards and informed consent detailed | **Yes**:  Two researchers independently coded transcripts, consensus meetings held | **Yes**:  Six themes clearly presented with quotations and summary tables | **Yes**:  Results offer actionable insights for PIFU implementation, identify potential barriers, and discuss transferability and study limitations |
| Lorenc 2022 (18) | **Yes**:  *“Objective … was to explore head and neck cancer patients' and their family members' views on acceptability and feasibility of patient-initiated follow-up (PIFU).”* | **Yes**:  An interpretive qualitative approach (semi-structured interviews with thematic analysis) was chosen to elicit subjective experiences, appropriate for the research aim. | **Yes**:  Serial interviews (30–60 min, online/phone) analysed with reflexive/­code-book thematic analysis match the exploratory aim. | **Yes**:  Purposive recruitment through three hospitals plus support/advocacy groups maximised variation in treatment stage and demographics; rationale is described. | **Yes**:  Interviews were audio-recorded, verbatim-transcribed, non-verbal notes taken, and topic guides piloted and refined; procedures clearly address the research question. | **Yes/Can't tell**: Interviewers’ non-clinical backgrounds and lack of prior relationship are noted, but there is limited reflection on how their positionality may have influenced data collection or interpretation | **Yes**:  Ethical approval from the North East Tyne & Wear South REC and HRA (ref 20/NE/0102); confidentiality safeguards and informed consent detailed | **Yes:**  Two researchers independently coded transcripts, consensus meetings held | **Yes:**  Themes are clearly presented with quotations and tables | **Yes**:  Findings generate practical recommendations for PIFU design and identify areas for further research |
| Moore 2022 (19) | **Yes**:  Purpose and rationale stated in abstract and introduction | **Yes**:  Research question concerns lived experience and pathway optimisation; authors justify interpretive approach | **Yes**:  Reflexive thematic analysis of individual interviews fits exploratory, experience-focused aim | **Yes**:  Purposive sampling across age, treatment and needs from two hospitals; rationale explicit | **Yes**:  20 audio-recorded interviews (25–60 min) with topic guide; reflexive diary kept; adaptations described | **Can’t tell**:  Interviewer background and lack of clinical role stated, and diary kept, but limited discussion of how role shaped analysis | **Yes**: University/NRES approvals, site R&D approval, written consent, confidentiality procedures detailed | **Yes**: Iterative inductive coding, double-coded transcripts, team consensus meetings, data saturation declared | **Yes**:  Five themes with quotations and a conceptual model; divergent views discussed | **Yes**:  Practical recommendations for service optimisation; transferability and limitations addressed |
| Drabarek 2023 (21) | **Yes**:  *“Aim … was to assess the acceptability of a hypothetical reduction in routinely scheduled visits … among patients with a history of localised melanoma.”* | **Yes**:  Mixed-methods study justified a qualitative content-analysis approach to explore subjective motivations and expectations. | **Yes**:  Inductive-plus-directed content analysis of open-ended survey responses at baseline and 6 months fits the exploratory aim. | **Yes**:  Of 100 randomized participants, 87 answered the questionnaire at baseline, 66 answered the questionnaire at 6 months,  and 79 provided a free-text explanation at either time point. | **Yes**:  Two open-ended questions delivered via REDCap; answers verbatim exported; three coders independently coded and resolved discrepancies by consensus. | **Yes**:  Coders’ roles (DA coded all data; two others cross-checked) are stated | **Yes**:  Sydney Local Health District HREC approval (2019/ETH13612) and electronic informed consent reported. | **Yes**:  Stepwise coding framework, triangulation by three analysts, consensus meetings, saturation checks, and frequency tables utilised | **Yes**:  Themes clearly presented | **Yes**:  Findings inform recruitment/retention strategies for the full MEL-SELF RCT and offer a transferable framework for other de-implementation studies |
| Jenkins 2023 (25) | **Yes**:  To highlight expectations and experience with PIFU including confidence in recognising and reporting BC-related symptoms, experiences of self-management and impact of COVID-19 pandemic | **Yes**:  The experiences of participants cannot be interpreted using a quantitative method | **Yes**:  Rationale for each aspect of the design (recruitment, sample size, data collection, analysis) were outlined and were reasonable/robust | **Yes**:  Full explanation of recruitment, rationale for recruitment and similarity in characteristics between those included vs those who chose not to participate | **Yes**:  Semi-structured interviews via telephone at 3, 6, 9 and 12 months with consistent interviewer | **Yes**:  Interviewers were independent of the recruiting hospital centres and location was neutral (over phone), no changes to research design evident | **Yes**:  Confidentiality discussed, and approval sought from ethics committee | **Yes**:  Thorough description of the process of analysis | **Yes**:  Findings clearly summarised | **Yes**:  Potential changes to current practice highlighted |
| Ackermann 2024 (29) | **Yes**:  *“Aim … was to determine motivators of participation and expectations … among patients enrolled in the MEL-SELF randomized clinical trial.”* | **Yes**:  Study uses qualitative content analysis of free-text survey answers to explore subjective motivations and expectations | **Yes**:  Inductive-plus-directed content analysis of open-ended baseline-survey responses aligns with the exploratory question. | **Yes**:  All first 100 randomized trial participants were invited; 98/100 (98 %) provided at least one free-text answer | **Yes**:  Two open-ended questions delivered in REDCap; responses exported, three coders independently coded, discrepancies resolved by consensus. | **Yes**:  Coders’ roles (DA coded all data; two others cross-checked) are stated | **Yes**:  Sydney Local Health District HREC approval (2019/ETH13612) and electronic informed consent reported. | **Yes**: Stepwise coding framework, triangulation by three analysts, consensus meetings, saturation checks, and frequency tables utilised | **Yes**:  Overarching themes and sub-themes are presented | **Yes**:  Findings used to refine recruitment and retention strategies in the ongoing MEL-SELF RCT and provide a transferable framework for other trials |
| Swartjes 2024 (31) | **Yes**:  *“…to investigate how patients experienced PHFU.”* | **Yes**:  Research question concerns subjective experience; qualitative approach explained | **Yes**:  Reflexive thematic analysis of semi-structured interviews matches exploratory aim | **Yes**:  Purposive sampling for variation in age, sex, tumour site, satisfaction. Rationale described. | **Yes**:  Interviews (median 33 min) audio recorded, verbatim-transcribed, guide piloted and refined | **Yes/Can't tell**: Interviewers’ credentials stated (medical trainees, no prior relationship) but limited discussion of how their positions might influence data | **Yes**:  Ethics committee approval and written informed consent reported | **Yes**:  Double-coding, consensus meetings after each transcript, investigator triangulation, audit trail. Participant quotations illustrate themes | **Yes**:  Themes clearly presented | **Yes**:  Practical implications (who PHFU suits, need for guidance) and limitations discussed; suggestions for service design given. |

**References**

1. Chadha K, Patrick T, Trowbridge S, Conway A, Waheed S. Open access follow up for breast cancer patients at East Surrey hospital: The way forward for breast cancer care. *Eur J Surg Oncol*. 2014;40(11):S72. doi:10.1016/j.ejso.2014.08.159
2. Li E, Rai S, Brown H, et al. If Patient Initiated Follow-Up and 5-year mammograms are in place, is routine annual follow-up also necessary for breast cancer patients after completing treatment? *Eur J Surg Oncol*. 2015;41(6):S63. doi:10.1016/j.ejso.2015.03.168
3. Batehup L, Porter K, Gage H, et al. Follow-up after curative treatment for colorectal cancer: longitudinal evaluation of patient initiated follow-up in the first 12 months. *Support Care Cancer*. 2017;25(7):2063-2073. doi:10.1007/s00520-017-3595-x
4. Kirshbaum MN, Dent J, Stephenson J, et al. Open access follow-up care for early breast cancer: a randomised controlled quality of life analysis. *Eur J Cancer Care (Engl)*. 2017;26(4):e12577. doi:10.1111/ecc.12577
5. Frankland J, Brodie H, Cooke D, et al. Follow-up care after treatment for prostate cancer: evaluation of a supported self-management and remote surveillance programme. *BMC Cancer*. 2019;19(1):368. doi:10.1186/s12885-019-5561-0
6. Hatem E, Tay LJ, Harvey H, Muir G, Brown C. Patient-led trial without catheter (TWOC) – is it feasible? *Br J Urol*. 2019;124(S3):81.
7. Jakobsen IH, Juul T, Thaysen HV, Johansen C, Laurberg S. Differences in baseline characteristics and 1-year psychological factors between participants and non-participants in the randomized, controlled trial regarding patient-led follow-up after rectal cancer (FURCA). *Acta Oncol*. 2019;58(5):5. doi:10.1080/0284186X.2019.1581948
8. Kitchen MO, Thursby H, Taylor M, Willard K, Mistry-Pain T. Self-Administered Intravesical Hyaluronic Acid Improves Symptoms and Quality of Life in a Patient-Centred Approach To Bladder Pain Syndrome Management. *J Endoluminal Endourol*. 2019;2(4):e1-e9. doi:10.22374/jeleu.v2i4.69
9. Walder E, Cox E. Evaluating patient attitudes to patient-led follow-up following breast cancer. *Eur J Surg Oncol*. 2020;46(6):e51. doi:10.1016/j.ejso.2020.03.192
10. Riis CL, Stie M, Bechmann T, et al. ePRO-based individual follow-up care for women treated for early breast cancer: impact on service use and workflows. *J Cancer Surviv*. 2021;15(4):485-496. doi:10.1007/s11764-020-00942-3
11. Jakobsen IH, Thaysen HV, Laurberg S, Johansen C, Juul T. Patient-led follow-up reduces outpatient doctor visits and improves patient satisfaction. One-year analysis of secondary outcomes in the randomised trial Follow-Up after Rectal CAncer (FURCA). *Acta Oncol*. 2021;60(9):9. doi:10.1080/0284186X.2021.195092
12. Ackermann DM, Dieng M, Medcalf E, et al. Assessing the Potential for Patient-led Surveillance After Treatment of Localized Melanoma (MEL-SELF): A Pilot Randomized Clinical Trial. *JAMA Dermatol*. 2022;158(1):33-42. doi:10.1001/jamadermatol.2021.4704
13. Balhorn J, Su’a B, Jin J, et al. Changing the routine: a move to patient initiated follow up to improve surgical outpatient clinic. *ANZ J Surg*. 2022;92(6):1394-1400. doi:10.1111/ans.17676
14. Drabarek D, Habgood E, Janda M, et al. Experiences of Patient-Led Surveillance, Including Patient-Performed Teledermoscopy, in the MEL-SELF Pilot Randomized Controlled Trial: Qualitative Interview Study. *JMIR Dermatol*. 2022;5(3):e35916. doi:10.2196/35916
15. Drabarek D, Habgood E, Ackermann D, et al. Perspectives and Experiences of Patient-Led Melanoma Surveillance Using Digital Technologies From Clinicians Involved in the MEL-SELF Pilot Randomized Controlled Trial: Qualitative Interview Study. *JMIR Dermatol*. 2022;5(4):e40623. doi:10.2196/40623
16. Katsura C, Tomouk T, Gathura E, Miranda B, Zweifel C. Improving Clinic DNA Rates with a Patient Initiated Follow Up Pathway: A Cohort Study. *Br J Surg*. 2022;109(Supplement_6):znac269.302. doi:10.1093/bjs/znac269.302
17. Lorenc A, Wells M, Fulton-Lieuw T, et al. Clinicians’ Views of Patient-initiated Follow-up in Head and Neck Cancer: a Qualitative Study to Inform the PETNECK2 Trial. *Clin Oncol*. 2022;34(4):230-240. doi:10.1016/j.clon.2021.11.010
18. Lorenc A, Greaves C, Duda J, et al. Exploring the views of patients’ and their family about patient-initiated follow-up in head and neck cancer: A mixed methods study. *Eur J Cancer Care (Engl)*. 2022;31(6):e13641. doi:10.1111/ecc.13641
19. Moore L, Matheson L, Brett J, et al. Optimising patient-initiated follow-up care – A qualitative analysis of women with breast cancer in the UK. *Eur J Oncol Nurs*. 2022;60. doi:10.1016/j.ejon.2022.102183
20. Stirling PHC, Simpson CJ, Ring D, Duckworth AD, McEachan JE. Virtual management of clinically suspected scaphoid fractures. *Bone Jt J*. 2022;104-B(6):709-714. doi:10.1302/0301-620X.104B6.BJJ-2021-1464.R2
21. Drabarek D, Ackermann D, Medcalf E, Bell KJL. Acceptability of a Hypothetical Reduction in Routinely Scheduled Clinic Visits Among Patients With History of a Localized Melanoma (MEL-SELF): Pilot Randomized Clinical Trial. *JMIR Dermatol*. 2023;6(1):e45865. doi:10.2196/45865
22. Hirniak J, Spurr S, Banerjee S. Introducing a Patient-Initiated Follow Up (PIFU) Pathway to a District General Hospital’s Urology Department: A Two-Cycle Quality Improvement Project Following the First 100 Patients. *Br J Surg*. 2023;110(Supplement_7):znad258.535. doi:10.1093/bjs/znad258.535
23. Hovdenak I, Thaysen HV, Bernstein IT, et al. Quality of life and symptom burden after rectal cancer surgery: a randomised controlled trial comparing patient-led versus standard follow-up. *J Cancer Surviv*. 2024;18(5):1709-1722. doi:10.1007/s11764-023-01410-4
24. Jenkins V, Matthews L, Solis-Trapala I, et al. Patients’ experiences of a suppoRted self-manAGeMent pAThway In breast Cancer (PRAGMATIC): quality of life and service use results. *Support Care Cancer*. 2023;31(10):570. doi:10.1007/s00520-023-08002-z
25. Jenkins V, Starkings R, Teoh M, et al. Patients’ views and experiences on the supported self-management/patient-initiated follow up pathway for breast cancer. *Support Care Cancer*. 2023;31(11):658. doi:10.1007/s00520-023-08115-5
26. Karlsen RV, Høeg BL, Dalton SO, et al. Are education and cohabitation associated with health-related quality of life and self-management during breast cancer follow-up? A longitudinal study. *Acta Oncol*. 2023;62(4):407-413. doi:10.1080/0284186X.2023.2199128
27. Abdul Gafoor SM, Robinson S, Diskantova S, et al. Patient-initiated follow-up for high-risk cutaneous squamous cell carcinoma: how we do it and 2 years of outcome data. *Clin Exp Dermatol*. 2024;49(10):1205-1212. doi:10.1093/ced/llae160
28. Abed H, Samson D, David M, Abed H, Samson D, David M. Early Discharge and Patient-Initiated Follow-Up in Hand Surgery: A New Norm Following Simple Hand Surgery? *Cureus*. 2024;16(1). doi:10.7759/cureus.52493
29. Ackermann D, Hersch J, Jordan D, et al. Participant Motivators and Expectations in the MEL-SELF Randomized Clinical Trial of Patient-Led Surveillance for Recurrent Melanoma: Content Analysis of Survey Responses. *JMIR Dermatol*. 2024;7(1):e58136. doi:10.2196/58136
30. Chaturvedi A, Russell H, Farrugia M, et al. Patient-directed follow-up for the clinical scaphoid fracture. *Bone Jt Open*. 2024;5(2):117-122. doi:10.1302/2633-1462.52.BJO-2023-0119.R1
31. Swartjes H, Aarts CJH, Deuning-Smit E, et al. Patient experiences with patient-led, home-based follow-up after curative treatment for colorectal cancer: a qualitative study. *BMJ Open*. 2024;14(2):e081655. doi:10.1136/bmjopen-2023-081655
32. Younis Z, Hamid MA, Khan MM, et al. Evaluation of Patient-Initiated Follow-Up (PIFU) Service in a Fracture Clinic: A Comprehensive Service Evaluation and Patient Satisfaction Audit. *Cureus*. 2024;16(11). doi:10.7759/cureus.73461
33. Fan KS, Haq I, Taha A, Carne A, Gademsetty C, Solan M. Efficient patient-initiated follow-up after orthopaedic injections. *Eur J Orthop Surg Traumatol*. 2025;35(1):77. doi:10.1007/s00590-025-04193-9
34. Naskar R, Garg V, Middleton E, Moverley R. The impact and utilisation of patient-initiated follow-ups (PIFU) in the elective upper-limb clinic at secondary care. *Ann R Coll Surg Engl*. Published online October 21, 2025. doi:10.1308/rcsann.2025.0073
